# Protein Kinase Activity of Phytochrome A Positively Correlates With Photoresponses in Arabidopsis

**DOI:** 10.3389/fpls.2021.706316

**Published:** 2021-07-30

**Authors:** Quyen T. N. Hoang, Jae-Yong Cho, Da-Min Choi, Ah-Young Shin, Jin A. Kim, Yun-Jeong Han, Jeong-Il Kim

**Affiliations:** ^1^Department of Integrative Food, Bioscience and Biotechnology, Chonnam National University, Gwangju, South Korea; ^2^Plant Systems Engineering Research Center, Korea Research Institute of Bioscience and Biotechnology, Daejeon, South Korea; ^3^National Academy of Agricultural Science, Rural Development Administration, Jeollabuk-do, South Korea; ^4^Kumho Life Science Laboratory, Chonnam National University, Gwangju, South Korea

**Keywords:** phytochromes, protein kinase, phytochrome-interacting factors, phosphorylation, protein degradation

## Abstract

Plant phytochromes are known as autophosphorylating serine/threonine protein kinases. However, the functional importance of their kinase activity is not fully elucidated. Previously, the kinase activity is shown to be necessary for the function of *Avena sativa* phytochrome A (AsphyA) using transgenic plants with mutants displaying reduced kinase activity, such as K411L and T418D. In this study, we isolated and analyzed two AsphyA mutants, K411R and T418V, that showed increased kinase activity. Transgenic *phyA-201* plants with these mutants showed hypersensitive responses to far-red (FR) light, such as shorter hypocotyls and more expanded cotyledons than those of control plant (i.e., transgenic *phyA-201* plant with wild-type AsphyA). Contrary to the mutants with reduced kinase activity, these mutants accelerated FR-induced phosphorylation and subsequent degradation of phytochrome-interacting factor 3 (PIF3) in Arabidopsis. Moreover, elongated hypocotyl 5 (HY5), a critical positive regulator of photoresponses in plants, accumulated in higher amounts in the transgenic plants under FR light than in the control plant. In addition, PIF1 degradation was accelerated in the transgenic plants. Consequently, the transgenic plants exhibit higher germination frequencies than the control plant. Collectively, our results demonstrate that the AsphyA mutants with increased kinase activity are hyperactive in plants, supporting a positive relationship between the kinase activity of phytochromes and photoresponses in plants.

## Introduction

Phytochromes are red (R) and far-red (FR) photoreceptors that regulate various plant photoresponses, such as seed germination and seedling de-etiolation (Legris et al., 2019; Tripathi et al., 2019). In higher plants, they are encoded by small gene families, for example, five members (phyA to phyE) in *Arabidopsis thaliana* (Mathews, 2010). Among them, phyA is light-labile and mediates FR light signaling, while phyB to phyE members are light-stable and play major roles in R light-mediated photomorphogenic development (Sharrock and Clack, 2002; Rausenberger et al., 2011). Phytochromes exist as either R light-absorbing Pr form or FR light-absorbing Pfr form, in which the inactive Pr form is converted to the physiologically active Pfr form upon exposure to light with red wavelength. This Pr-to-Pfr photoactivation induces a highly regulated signaling network for plant photomorphogenesis, including the translocation of phytochromes from the cytosol into the nucleus and their interactions with a wide array of signaling partners (Bae and Choi, 2008; Jing and Lin, 2020).

Among the interacting partners, PHYTOCHROME INTERACTING FACTORs (PIFs) play the central roles in phytochrome-mediated light signaling (Leivar and Quail, 2011). For example, among the eight PIFs (PIF1 to PIF8) in *A. thaliana*, PIF3 promotes and maintains skotomorphogenic development of etiolated seedlings in darkness by repressing photomorphogenesis, and PIF1 plays a crucial role in inhibiting seed germination in the dark (Pham et al., 2018a; Favero, 2020). A previous study on the signaling transduction mechanism from phytochromes to PIFs showed PIF3 to be phosphorylated and subsequently degraded in plants via the ubiquitin/26S proteasome pathway in a phytochrome-dependent manner (Al-Sady et al., 2006). In addition, phytochromes are also known to inhibit the PIF activity through sequestration (Park et al., 2018). Thus, the photoactivated phytochromes inhibit PIF3 function in plants by inducing their degradation and sequestration. Other PIFs, such as PIF1, PIF4, PIF5, and PIF7, are also shown to be regulated by phosphorylation in plants (Shen et al., 2007, 2008; Lorrain et al., 2008; Huang et al., 2018). In particular, PIF1 is rapidly phosphorylated under FR and R light before being degraded, in which phyA plays a dominant role in regulating the PIF1 degradation following initial light exposure (Shen et al., 2008). In addition to this regulation, phytochromes inhibit the activity of CONSTITUTIVE PHOTOMORPHOGENIC 1 (COP1) and SUPPRESSOR OF PHYA-105 (SPA) complex that functions as an E3 ligase for photomorphogenesis-promoting transcription factors, especially ELONGATED HYPOCOTYL 5 (HY5) (Lu et al., 2015; Sheerin et al., 2015; Podolec and Ulm, 2018). As a result, the photoactivated phytochromes can induce HY5 accumulation for photomorphogenic development. Therefore, a principal regulatory mechanism of phytochromes is proposed as the transcriptional regulation of photoresponsive genes via removal of negative regulators such as PIFs and accumulation of positive regulators such as HY5.

Phytochromes have been suggested as autophosphorylating serine/threonine kinases (Yeh and Lagarias, 1998; Han et al., 2010). In a previous study, we obtained *Avena sativa* phytochrome A (AsphyA) mutants displaying reduced kinase activity, such as K411L and T418D, and showed that the transgenic plants with these mutants exhibited hyposensitive responses to FR light (Shin et al., 2016). In the same study, we confirmed that plant phytochromes directly phosphorylate PIFs *in vitro*, in which the K411L and T418D mutants exhibited significantly reduced phosphorylation of PIF3. Accordingly, FR light-induced phosphorylation and protein degradation of PIF3 are significantly prevented in the transgenic plants, thereby proposing a positive relationship between the phytochrome’s kinase activity and PIF3 phosphorylation (Shin et al., 2016). Later, other kinases have also shown to phosphorylate PIF3, such as PHOTOREGULATORY PROTEIN KINASEs (PPKs) and BRASSINOSTEROID INSENSITIVE 2 (BIN2) (Ling et al., 2017; Ni et al., 2017). In addition, CASEIN KINASE 2 (CK2) was previously shown to phosphorylate PIF1 at multiple sites, and the PIF1 degradation rate of phosphorylation site mutants was significantly reduced in plants (Bu et al., 2011). More recently, SPA1 was reported to act as a serine/threonine kinase that directly phosphorylates PIF1, which was necessary for the light-induced phosphorylation and subsequent degradation (Paik et al., 2019). Therefore, phosphorylation may be a prerequisite for the 26S proteasome-mediated degradation of PIF3 and PIF1, which is an important step for the initiation of photomorphogenic development (Li et al., 2011; Hoang et al., 2019).

In our previous study, we confirmed the protein kinase activity of phytochromes on PIFs, including PIF3 and PIF1 (Shin et al., 2016). In addition, we obtained AsphyA mutants with reduced kinase activity, such as K411L and T418D, and demonstrated their reduced phyA function in transgenic plants. However, the functional importance of phytochrome kinase activity was not fully elucidated. Thus, in the present study, we extended our work by obtaining and analyzing the AsphyA mutants showing increased kinase activity. Using the kinase activity assays of site-mutants of AsphyA, we obtained two mutants, K411R and T418V, that showed a higher kinase activity on PIF3 than wild-type AsphyA. Then, we generated transgenic *phyA-201* plants with the mutants and demonstrated their enhanced responses to FR light, confirming the positive relationship between the kinase activity of AsphyA and photoresponses in plants. Moreover, we analyzed the kinase activity of AsphyA mutants on PIF1, and investigated PIF1-mediated inhibition of seed germination using the transgenic plants. Overall, the present study provides further evidence that the kinase activity of phytochromes is important for the removal of PIFs, the negative regulators of photomorphogenesis, to mediate plant light signaling.

## Materials and Methods

### Preparations of Recombinant Proteins

The QuickChange^TM^ site-directed mutagenesis kit (Agilent Technologies) was used to generate AsphyA mutants (K411E, K411R, and T418V) using the mutagenic primers listed in Supplementary Table 1. In this study, we also included E410Q, K411L, and T418D mutants used in our previous study (Shin et al., 2016). Full-length recombinant proteins of AsphyA, with a ten-amino acid streptavidin affinity-tag (SAWRHPQFGG; strep-tag) at the C-terminus, were expressed and purified using the *Pichia pastoris* protein expression system (Thermo Fisher Scientific) and streptavidin affinity chromatography (IBA) as described previously (Han et al., 2019). Phycocyanobilin (PCB) was added to the final concentration of 20 μM as a chromophore before purification under dim green light. The purified Pr form of AsphyA was exposed to R light to generate the Pfr form, which was confirmed using a diode array UV/VIS spectrophotometer (Cary). A zinc fluorescence assay was performed to confirm the ligation of PCB in AsphyA proteins and differential spectra (ΔAbsorbance) were obtained by subtracting the Pfr absorption spectrum from the Pr absorption spectrum, as described previously (Shin et al., 2016; Han et al., 2019).

Full-length recombinant proteins of PIF3, PIF1, FAR-RED ELONGATED HYPOCOTYL 1 (FHY1), and FHY1-LIKE (FHL) were prepared using the methods reported previously (Jeong et al., 2016; Shin et al., 2016). Their genes were subcloned into the pGEX 4T vector (GE Healthcare) with glutathione S-transferase and streptavidin (GST/strep) affinity tags at the N- and C-termini, respectively. The *E. coli* strain BL21-CodonPlus^TM^ (Agilent Technologies) was used for the expression and the GST/strep-tagged recombinant proteins were purified by the streptavidin affinity chromatography.

### Phytochrome Kinase Assay

A reaction mixture (20 μL), containing 1.0 μg of full-length AsphyA (as the Pfr form) and 1.0 μg of GST/strep-fused PIF3 (as substrate), was prepared in a kinase buffer (25 mM Tris-HCl, pH 7.8, 0.2 mM EDTA, 4 mM DTT, and 5 mM MgCl_2_), as described previously (Shin et al., 2016). The reactions were started by adding 150 μM ATP containing 10 μCi of [γ-^32^P] ATP and incubated at 30°C for 1 h. Proteins were then resolved on SDS-PAGE gels and dried under vacuum before autoradiography by exposing on x-ray films. Coomassie blue staining and zinc fluorescence assay were performed before the drying to verify the AsphyA proteins.

### Photoaffinity Labeling of AsphyA With an Azido-ATP Analog

Photoaffinity labeling of AsphyA with 2-N_3_-ATP-biotin-long chain-hydrazone (2-azido-ATP; Affinity Labeling Technologies, Inc) was performed using the method reported previously (Shin et al., 2016). Briefly, 2.0 μg of purified AsphyA protein (as the Pfr form) was preincubated in 50 μL of a photoaffinity labeling buffer (20 mM Tris-HCl, pH 7.0, 2 mM EDTA, 2 mM MgCl_2_, and 150 μM ATP) for 30 min on ice, and 2-azido-ATP was added in various amounts (1, 5, 10, 20, 50, and 100 μM). After 5 min of additional incubation on ice, sample was irradiated with the UV light (254 nm) for 90 s. The reactions were then immediately stopped by adding SDS sample buffer, and the 2-azido-ATP-labeled AsphyA proteins were detected using 1:2,000 avidin-HRP (A-115; Boston Biochem, Inc). The developed images were scanned by ImageJ to quantitate the photoaffinity labeling. The 2-azido-ATP labeled signals were normalized to AsphyA protein levels using the zinc fluorescence intensities. The percentage of 2-azido-ATP labeling was calculated by assuming the labeling of wild-type AsphyA with 100 μM 2-azido-ATP as 100%.

### *In vitro* Protein-Protein Interaction Assay

Pull-down experiments were performed to examine protein-protein interaction between AsphyA and phytochrome-interacting proteins (PIF3, PIF1, FHY1, and FHL), as described previously (Jeong et al., 2016; Shin et al., 2016). A total of 2 μg of AsphyA protein (either the Pr or Pfr form) and 2 μg of phytochrome-interacting proteins with GST/strep-tag were mixed and incubated at 4°C for 60 min in 1 mL of pull-down buffer (100 mM Tris-HCl, pH 7.8, 1 mM EDTA, 150 mM NaCl, and 100 μg⋅mL^–1^ BSA) with gentle rotation. Next, 50 μL of glutathione resin was added and incubated for another 30 min. After washing, AsphyA and GST/strep-fused phytochrome-interacting proteins were detected with 1:5,000 AsphyA-specific (oat25) and 1:2,000 GST-specific (sc-138; Santa Cruz Biotechnology) monoclonal antibodies, respectively.

### Plant Materials Used in This Study

pBI121 vectors harboring cDNAs of *AsPHYA* mutant genes (K411E, K411R, and T418V) were introduced into phyA-deficient Arabidopsis (*phyA-201*; L*er* ecotype). After obtaining homozygous lines, western blot analysis was performed with crude extracts obtained from 4-d-old dark-grown seedlings to examine the protein expression levels using AsphyA-specific (oat25) monoclonal antibody. For loading controls, Arabidopsis translationally controlled tumor protein (TCTP; At3g16640) was detected with 1:10,000 TCTP-specific polyclonal antibody (Kim et al., 2012). We also included transgenic plants expressing wild-type AsphyA (AsA-OX), K411L, or T418D. Moreover, to analyze nuclear localization, enhanced green fluorescent protein (eGFP)-fused K411R and T418V genes were subcloned into pBI121, which were used to transform *phyA-201*. As a control, we used a transgenic plant expressing eGFP-fused wild-type AsphyA reported previously (Jeong et al., 2016).

Transgenic plants co-expressing AsphyA and eGFP-fused PIF3 (PIF3:eGFP) were also generated to investigate *in vivo* PIF3 phosphorylation and degradation. For this, the pCAMBIA3300-eGFP vector harboring *PIF3* was introduced into the transgenic plants with K411R and T418V (named K411R/PIF3 and T418V/PIF3). As controls, we included transgenic L*er* and *phyA-201* plants expressing PIF3:eGFP (named L*er*/PIF3 and *phyA-201*/PIF3), as well as the transgenic plant co-expressing wild-type AsphyA and PIF3:eGFP (named AsA-OX/PIF3) used previously (Shin et al., 2016).

### Photoresponse and Phenotypic Analyses

After sterilized seeds were stratified at 4°C for 3 days in the dark, they were sown on 0.6% phytoagar plates containing half-strength MS salts and vitamins. The seeds were then exposed to white light (100 μmol⋅m^–2^⋅s^–1^) for 8 h to promote germination, returned to darkness at 21°C for 1 day, and grown further for 3.5 days in the dark or under continuous far-red (cFR) light with various fluence rates (0.01, 0.1, 1, and 10 μmol⋅m^–2^⋅s^–1^), in an LED growth chamber (Vision Science, South Korea; for FR, λ_*max*_ = 738 nm and bandwidth = 42 nm). Hypocotyl lengths and cotyledon areas and angles were measured from the pictures of seedlings using ImageJ.

For the phenotypic analysis of mature plants, Arabidopsis plants were grown at 21°C in a long day condition (i.e., 16 h light/8 h dark photoperiod). Plant height was measured from 4-week-old plants, and flowering time was estimated from the days at bolting. Thirty plants of each line were used for these measurements.

### Anthocyanin and Chlorophyll Content Analysis

The anthocyanin and total chlorophyll content of seedlings were determined as described (Fankhauser and Casal, 2004). Briefly, 50 seedlings from each line were collected, ground into fine powder in liquid nitrogen, and incubated overnight in 500 μL of methanol acidified with 1% HCl by shaking in the dark. Next, 500 μL of chloroform was added for extraction, and the anthocyanin content was estimated by subtracting A_657_ from A_530_ of the aqueous phase, which was determined using a UV/VIS spectrophotometer (Cary). Chlorophylls were extracted by incubating the ground samples in 1 mL of 80% acetone, with overnight shaking in the dark. Total chlorophyll content was estimated using the equation, chlorophyll_*a*__+__*b*_ = 7.15 × A_660_ +18.71 × A_647_.

### Confocal Microscopy Analysis

To investigate nuclear localization of the kinase mutants (K411R and T418V), transgenic *phyA-201* plants expressing eGFP-fused AsphyA constructs were used. Four-day-old dark-grown seedlings were either kept in the dark or exposed to white light (100 μmol⋅m^–2^⋅s^–1^) for 5 min before the subcellular localization analysis. For analyzing GFP fluorescence, seedlings were transferred onto a microscope slide, covered with a cover slip, and observed using a laser scanning confocal microscope (Leica TCS SP5 AOBS/Tandem) at the Gwangju Center of Korea Basic Science Institute (KBSI).

### Phytochrome Degradation Assay

Four-day-old dark-grown seedlings were exposed to continuous white light (150 μmol⋅m^–2^⋅s^–1^) for the indicated durations (3, 6, 12, and 24 h), before collecting samples. The samples (∼50 seedlings) were frozen in liquid nitrogen and ground using TissueRuptor (Qiagen) in 200 μL of extraction buffer [70 mM Tris-HCl, pH 8.3, 35% ethylene glycol, 98 mM (NH_4_)_2_SO_4_, 7 mM EDTA, 14 mM sodium metabisulfite, 0.07% polyethyleneimine, and protease inhibitors (Roche)]. After quantifying protein concentrations by Qubit assays (Thermo Fisher Scientific), total protein extract (60 μg) was subjected to western blot analysis and AsphyA was detected using oat25 monoclonal antibody (1:5,000). TCTP proteins in the same samples were also detected for loading controls.

### *In vivo* Phosphorylation and Degradation of PIF3 and PIF1

To investigate the light-induced protein degradation of PIF3, 4-d-old dark-grown seedlings were kept in the dark or exposed to either R (10 μmol⋅m^–2^⋅s^–1^) or FR (5 μmol⋅m^–2^⋅s^–1^) light for 5 min before collecting samples for protein extraction. For the time-dependent degradation of PIF3 under FR light, 4-d-old dark-grown seedlings were kept in the dark or exposed to FR (5 μmol⋅m^–2^⋅s^–1^) for the time indicated (5, 10, and 15 min). To detect light-induced phosphorylation of PIF3 more clearly, 4-d-old dark-grown seedlings of transgenic *phyA-201* plants co-expressing AsphyA and eGFP-fused PIF3 (AsA-OX/PIF3, K411R/PIF3, and T418V/PIF3) were exposed to pulsed FR (FRp) light for 1 min (6,000 μmol⋅m^–2^) or 2 min (12,000 μmol⋅m^–2^), and incubated further in the dark for 5 min. After collecting the seedling samples, proteins were extracted immediately using a buffer (100 mM Tris-HCl, pH 7.8, 4 M urea, and protease inhibitors) and each extracted protein (60 μg) was separated on 6% SDS-polyacrylamide gel. The PIF3 protein levels in plants were then determined by western blotting using 1:3,000 PIF3-specific polyclonal antibody (Choi et al., 2021) or 1:500 GFP-specific monoclonal antibody (sc-9996; Santa Cruz Biotechnology).

To investigate the light-induced degradation of PIF1 protein, 4-d-old dark-grown seedlings were kept in the dark or exposed either to R (20 μmol⋅m^–2^⋅s^–1^) or FR (20 μmol⋅m^–2^⋅s^–1^) light for 5 min before collecting samples for protein extraction. In this experiment, not only transgenic plants of two AsphyA mutants with higher kinase activity (K411R and T418V), but also those of two mutants with lower kinase activity (K411L and T418D) used previously (Shin et al., 2016), were included with AsA-OX as a control. For the time-dependent degradation of PIF1 under FR light, 4-d-old dark-grown seedlings were kept in the dark or exposed to FR (20 μmol⋅m^–2^⋅s^–1^) for the time indicated (5, 10, and 30 min). The PIF1 levels were analyzed by western blotting using PIF1-specific polyclonal antibody (1:1,000) produced by the same method used for the PIF3-specific antibody (Choi et al., 2021).

### Quantitative PCR and HY5 Accumulation Analyses

For FR-responsive gene expression analysis, 3-d-old dark-grown seedlings were exposed to FR (10 μmol⋅m^–2^⋅s^–1^) light for 1 h, before harvesting. Samples were immediately frozen with liquid nitrogen and total RNA was isolated using RNAiso Plus (TaKaRa) and cleaned up using RNeasy mini kit (Qiagen). After cDNA synthesis using cDNA EcoDry Premix^TM^ (TaKaRa), the expression of *PRR9* (pseudo-response regulator 9) and *HY5* was analyzed by quantitative real-time PCR analysis using Stratagene Mx3005P with Brilliant III Ultra-Fast SYBR Green Q-PCR Master Mix (Agilent Technologies), using the corresponding primer pairs (Supplementary Table 1). *ACT2* expression was used for data normalization, and the relative expression levels were estimated by setting the transcript levels in L*er* as 1.

For analyzing HY5 accumulation in response to FR light, seedlings were grown for 4 days under continuous FR (0.01 or 5 μmol⋅m^–2^⋅s^–1^), before samples were collected. Total proteins were extracted using a lysis buffer [50 mM Tris, pH 7.5, 150 mM NaCl, 1 mM EDTA, 10 mM sodium fluoride, 25 mM β-glycerophosphate, 2 mM sodium orthovanadate, 10% glycerol, 0.1% Tween 20, 1 mm dithiothreitol, 1 mM phenylmethylsulfonyl fluoride, and 1× complete protease inhibitor cocktail (Sigma-Aldrich)]. Each extracted protein sample (60 μg) was separated on 10% SDS-polyacrylamide gel, and HY5 was detected by western blotting using 1:1,000 HY5-specific polyclonal antibody (R1245-1b; Abiocode). In this analysis, *hy5* and *cop1* mutants were included as negative and positive controls, respectively. Relative intensities of HY5 protein were estimated from the blots using ImageJ, assuming the HY5 level in L*er* as 1. The band intensity of TCTP was used for normalization.

### Seed Germination Assay

In this study, phyA-dependent seed germination was analyzed using the protocol reported previously (Song and Choi, 2019), with some modifications. Sterilized and plated seeds were irradiated with FRp (5 μmol⋅m^–2^⋅s^–1^ for 5 min) and incubated at 21°C for 2 days in the dark. Seeds were then exposed with FR fluence rates of 0.5 or 5 μmol⋅m^–2^⋅s^–1^ for the time indicated (0, 5, 15, 30, 60, and 240 min), and further incubated for 3 days either in the dark or under white light (WL, 150 μmol⋅m^–2^⋅s^–1^). Germination percentages were calculated from the number of germinated seeds per total number of plated seeds. In addition to L*er*, *phyA-201* and AsA-OX, Col-0 and *pif1* (Col-0 background) plants were included as controls.

### Statistical Analysis

Experimental data were subjected to analysis of variance (ANOVA) using IBM SPSS Statistics 20 software. Significant difference of mean values was compared by the LSD at *P* < 0.05 (labeled “^∗^’) and *P* < 0.01 (labeled “^∗∗^”). All of the data were represented as the mean ± SD of at least three independent experiments.

## Results

### AsphyA Mutants With Increased Kinase Activity

**FIGURE 1:**
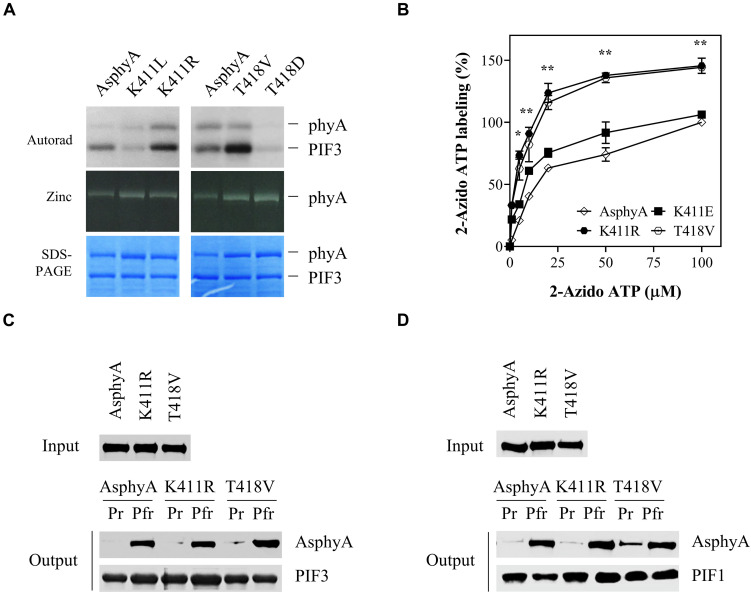
*Avena sativa* phytochrome A (AsphyA) mutants with increased kinase activity. **(A)** Kinase activity assays of AsphyA mutants with phytochrome-interacting factor 3 (PIF3) as a substrate. 1.0 μg of GST/strep-fused PIF3 (∼0.3 μM) was added in reaction mixtures with 1.0 μg of full-length AsphyA protein (∼0.2 μM; Pfr form). Autoradiograms (top), zinc fluorescence images (middle), and SDS-PAGE gels (bottom) are shown. **(B)** ATP-binding affinity assays of AsphyA mutants using photoaffinity labeling with 2-N_3_-ATP-biotin-long chain-hydrazone (2-azido-ATP). A total of 2.0 μg of full-length AsphyA protein (∼0.16 μM) was labeled with the indicated concentrations of 2-azido-ATP. The percentages of 2-azido-ATP labeling were obtained assuming the wild-type AsphyA labeling with 100 μM azido-ATP as 100%. Data represent means ± SD from three independent measurements. Significant changes in comparison to wild-type AsphyA are indicated (*P < 0.05 and **P < 0.01, as determined using Tukey’s test). **(C,D)** *In vitro* protein–protein interaction analyses of AsphyA mutants with PIF3 and PIF1. A total of 2 μg of full-length AsphyA (either Pr or Pfr form) was incubated with 2 μg of GST/strep-fused PIF3 **(C)** or PIF1 **(D)** at 4°C for 60 min. Glutathione bead-bound proteins were then pelleted and analyzed by western blotting with anti-AsphyA (oat25) or anti-GST (sc-138) monoclonal antibodies.

Previously, we provided evidence that phytochromes function as protein kinases in plant light signaling by showing that transgenic plants with AsphyA mutants displaying reduced kinase activity exhibited hyposensitive responses to FR light (Shin et al., 2016). In that study, we mutagenized amino acid residues in a putative ATP binding site (403–426 aa) and obtained AsphyA mutants showing a reduced binding affinity to ATP, such as K411L and T418D. In this study, we further mutagenized these sites to obtain AsphyA mutants with increased kinase activity. Consequently, we isolated two mutants, K411R and T418V, with a higher kinase activity than wild-type AsphyA (Figure 1A and Supplementary Figure 1). The protein kinase activity assays in a time-dependent manner showed an increase in PIF3 phosphorylation by the K411R and T418V mutants, compared with that by wild-type AsphyA (Supplementary Figure 2A). In contrast, the K411L and T418D mutants exhibited a reduced PIF3 phosphorylation, as reported previously (Shin et al., 2016). Analysis of the spectroscopic properties of the K411R and T418V mutants using purified recombinant proteins showed normal chromophore (i.e., PCB) ligation and Pr and Pfr absorption spectra similar to those of wild-type AsphyA (Supplementary Figure 1). In this study, we also included K411E mutant as another control that exhibited kinase activity similar to wild-type AsphyA (Supplementary Figure 2B). Notably, compared to wild-type AsphyA, the K411E mutant displayed blue-shifted Pr (651 vs. 654 nm) and Pfr (716 vs. 720 nm) absorption peaks (Supplementary Figure 1). Thus, we successfully obtained two AsphyA mutants that exhibited normal spectroscopic properties, including Pr-to-Pfr phototransformation, but displayed a higher kinase activity than wild-type AsphyA.

To account for the increased kinase activity of the K411R and T418V mutants, photoaffinity labeling experiments with 2-azido-ATP were conducted to investigate their binding affinity to ATP. The results showed that the ATP-binding affinity increased significantly (∼1.5 fold) in both K411R and T418V mutants compared with that in wild-type AsphyA and K411E (Figure 1B and Supplementary Figure 3). Furthermore, we examined the protein-protein interactions of the AsphyA proteins with substrate proteins, PIF3 and PIF1, which confirmed that the K411R and T418V mutants interacted with PIF3 and PIF1 in a Pfr-specific manner, similar to wild-type AsphyA (Figures 1C,D). Collectively, these results indicate that the K411R and T418V mutants obtained in this study exhibit normal spectroscopic and PIF interaction properties, but a higher binding affinity to ATP, resulting in the increased kinase activity.

### Photoresponses of Transgenic Plants Expressing the AsphyA Mutants With Increased Kinase Activity

**FIGURE 2:**
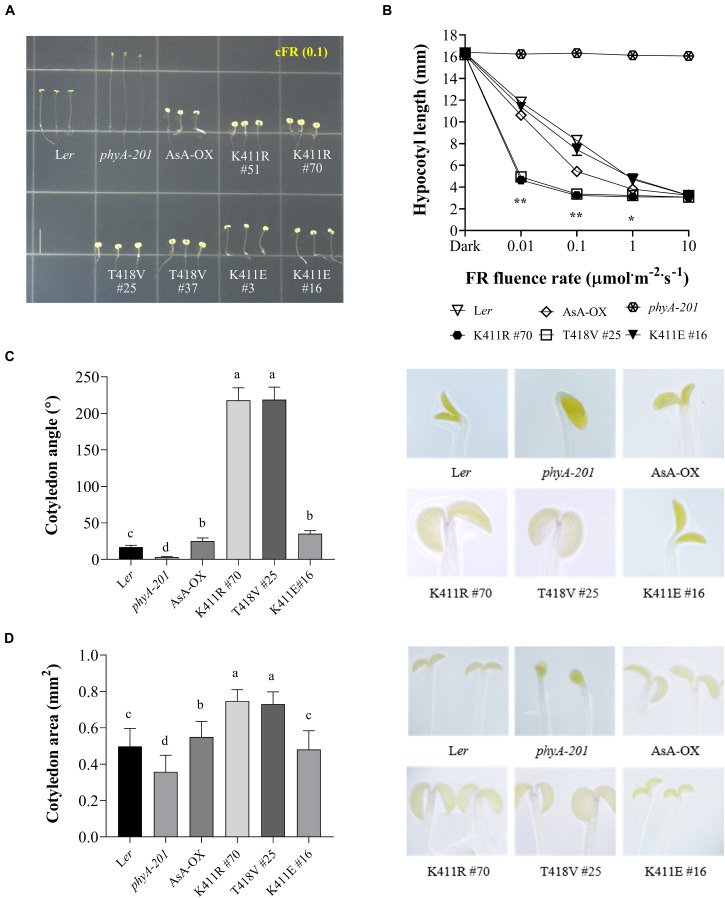
Far-red photoresponse analyses of transgenic *phyA-201* plants with AsphyA kinase mutants. **(A)** Hypocotyl de-etiolation of representative 4.5-days-old seedlings grown under continuous far-red (cFR, 0.1 μmol⋅m^–2^⋅s^–1^) light. L*er*, wild-type Arabidopsis; *phyA-201*, phyA-deficient Arabidopsis (L*er* ecotype); AsA-OX, transgenic *phyA-201* with wild-type AsphyA; K411R, T418V, and K411E, transgenic *phyA-201* lines with the corresponding AsphyA mutants. Scale bar = 5.0 mm. **(B)** FR fluence rate-response curves for the inhibition of hypocotyl growth. Data represent means ± SD (*n* ≥ 30). Significant changes in comparison to AsA-OX are indicated (*P < 0.05 and **P < 0.01, Tukey’s test). **(C,D)** Cotyledon angles and areas of 4.5-days-old seedlings under 0.01 or 0.1 μmol⋅m^–2^⋅s^–1^ of cFR light, respectively. Cotyledon angles **(C)** and areas **(D)** were measured from the seedling images using ImageJ. Data are means ± SD (*n* ≥ 30). Means with different letters are significantly different at *P* < 0.01, using Duncan’s multiple range test.

To investigate the function of the AsphyA mutants *in vivo*, we generated transgenic plants (*phyA-201* background) expressing K411R, T418V, and K411E under the control of the 35S promoter (Supplementary Figure 4). As phytochrome function exhibits a strong dependency on its level of expression, western blotting was performed to select transgenic lines showing expression levels of AsphyA protein comparable to those of the transgenic line with wild-type AsphyA (AsA-OX) used in our previous study (Shin et al., 2016). Then, we investigated the seedling de-etiolation responses under cFR light, because it is well-known that phyA participates exclusively in the FR-induced inhibition of hypocotyl elongation (Fankhauser and Casal, 2004). Hypocotyl lengths of the transgenic plants expressing the K411R and T418V mutants were notably shorter than those of both wild-type Arabidopsis (L*er*) and AsA-OX plants (Figure 2A and Supplementary Figure 4). However, there was no significant difference in the hypocotyl lengths between K411R and T418V transgenic plants, suggesting that the K411R and T418V mutants are similarly hyperactive in plants. In the case of the K411E transgenic plants, the hypocotyl lengths were comparable to those in L*er*, but longer than in AsA-OX, suggesting a reduced activity of the mutant in comparison to wild-type AsphyA. Furthermore, FR fluence rate-response curves for the inhibition of hypocotyl growth confirmed that transgenic seedlings with K411R and T418V were more responsive to FR than those of control plants (i.e., L*er* or AsA-OX), whereas the K411E seedlings were less responsive than AsA-OX (Figure 2B). Moreover, transgenic seedlings with K411R and T418V showed significantly enhanced cotyledon opening and expansion under cFR (Figures 2C,D). Collectively, these results suggest that the AsphyA mutants with increased kinase activity are hyperactive in plants, thus increasing the sensitivity of the seedlings to FR light.

**FIGURE 3:**
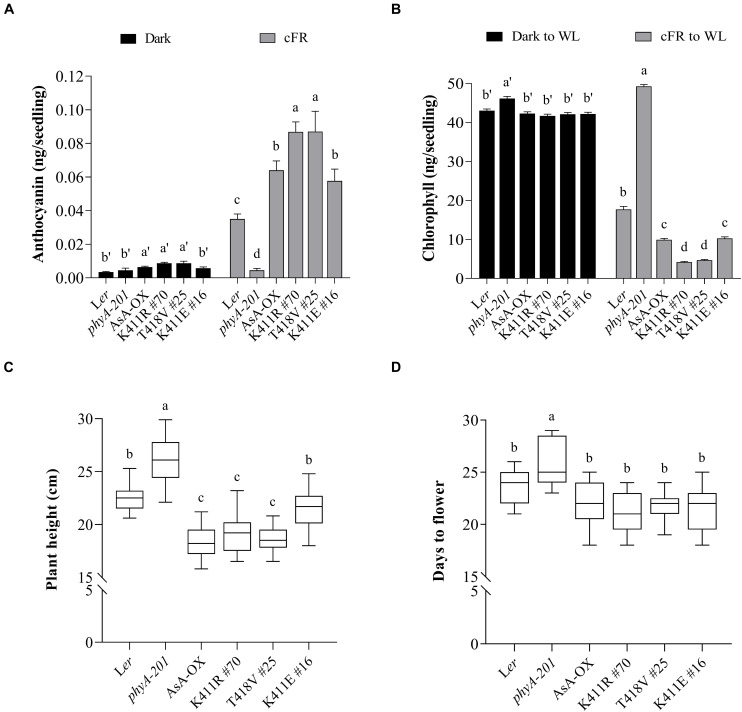
Pigment and phenotypic analysis of transgenic plants with AsphyA kinase mutants. **(A)** Anthocyanin content analysis. Fifty seedlings grown for 4 days in the dark or under cFR (5 μmol⋅m^–2^⋅s^–1^) were used for the extraction of samples. Data represent means ± SD from three independent measurements. **(B)** Chlorophyll content analysis. Fifty seedlings grown for 3 days in the dark or under cFR (5 μmol⋅m^–2^⋅s^–1^) were transferred to white light (WL, 150 μmol⋅m^–2^⋅s^–1^) and grown further for 1 days before analysis. Data represent means ± SD from three independent measurements. **(C,D)** Phenotypic analysis. Plant height **(C)** was measured from 4-week-grown plants, and flowering time **(D)** was measured from the days at bolting, with plants grown under long day condition (16 h light/8 h dark photoperiod). Data represent means ± SD (*n* ≥ 30). Means with different letters are significantly different at *P* < 0.01, using Duncan’s multiple range test.

In addition to regulating hypocotyl growth and cotyledon development, phyA also mediates various plant responses to FR light. For example, anthocyanin accumulation in response to light is reportedly induced by phyA function (Shin et al., 2007; Seaton et al., 2018). Thus, we compared the anthocyanin content in cFR-grown seedlings, and found that the K411R and T418V plants showed higher anthocyanin accumulation than L*er* and AsA-OX control plants (Figure 3A). Furthermore, using cFR-grown seedlings, we investigated the blocking of greening under WL, which is known to be mediated by phyA (Barnes et al., 1996). As controls, we observed that phyA-deficient plant (*phyA-201*) did not show the inhibition of light-induced greening, in which the chlorophyll content of the “cFR to WL” seedlings was similar to that of the “Dark to WL” seedlings (Figure 3B). In contrast, L*er* showed the blocking of greening (i.e., photobleached phenotype), resulting in a significantly reduced chlorophyll content in the ‘cFR to WL’ seedlings. In the case of K411R and T418V plants, greater inhibition of WL-induced greening was observed in the ‘cFR to WL’ seedlings than in L*er* and AsA-OX plants (Figure 3B). Notably, the K411E plant showed anthocyanin and chlorophyll levels comparable to those of AsA-OX. As the FR-mediated blocking of greening is dependent on phyA function, these results further suggest that the AsphyA mutants with increased kinase activity are hyperactive in plants.

We also investigated adult phenotypes, such as plant height and flowering time, because phyA is known to act as a weak repressor of elongated growth and flowering (Legris et al., 2019). Analysis of the plants grown in a long day condition showed that the heights of the K411R and T418V plants were less than those of L*er* and K411E plants, but comparable to those of AsA-OX (Figure 3C). Regarding flowering time, the transgenic plants with all AsphyA constructs used in this study exhibited a slight early flowering compared to L*er*, although the difference was not significant (Figure 3D). These results indicate that the transgenic plants of the AsphyA mutants with increased kinase activity display similar plant height and flowering time to those of AsA-OX.

### FR Light-Induced Degradation and Phosphorylation of PIF3 in Transgenic Plants

To account for the increased function of the K411R and T418V mutants in plants, we examined light-dependent nuclear localization and protein degradation, because these characteristics are known to affect the *in vivo* function of phyA. First, we investigated the interaction of AsphyA with FHY1 and FHL that are required for phyA nuclear localization (Hiltbrunner et al., 2005; Rosler et al., 2007). Results showed that the K411R and T418V mutants interacted with FHY1 and FHL, as in wild-type AsphyA (Supplementary Figure 5A). Second, we further analyzed light-dependent nuclear localization using transgenic *phyA-201* plants with eGFP fusion constructs and found that both mutants localized in a similar manner as wild-type AsphyA under the same light conditions (Supplementary Figure 5B). When we measured the ratios of nuclear and cytoplasmic signals, similar ratios (around 80.5–81.5% in the nucleus and 18.5–19.5% in the cytoplasm) were observed in transgenic plants with eGFP-fused AsphyA and the mutants (Supplementary Table 2). Third, we investigated light-dependent degradation of phyA protein and found that the K411R and T418V mutants degraded faster than wild-type AsphyA (Supplementary Figure 6). Therefore, we ruled out nucleus localization and protein stability as the reason for the hyperactivity of the mutants.

**FIGURE 4:**
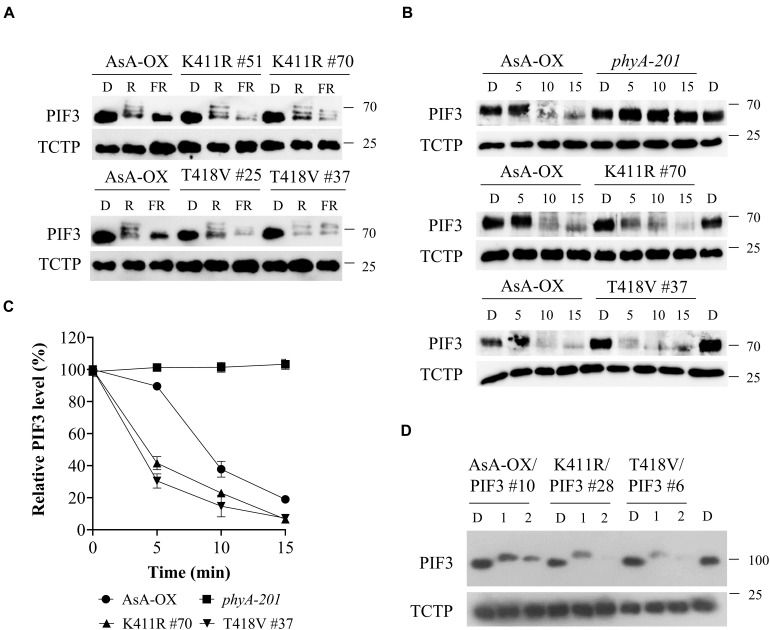
FR light-induced phosphorylation and degradation of PIF3 in transgenic plants. **(A)** PIF3 degradation in transgenic plants expressing AsphyA under different light conditions. Four-day-old dark-grown seedlings were kept in the dark (D) or exposed to either red (R, 10 μmol⋅m^–2^⋅s^–1^) or far-red (FR, 5 μmol⋅m^–2^⋅s^–1^) light for 5 min before collecting samples for protein extraction. PIF3 protein was detected by western blotting using PIF3-specific antibody, and loading controls (TCTP) were shown in the lower panels. **(B)** Time-dependent degradation of PIF3 under FR light. Four-day-old dark-grown seedlings were kept in the dark (D) or exposed to FR (5 μmol⋅m^–2^⋅s^–1^) for the time indicated (5, 10, and 15 min), and PIF3-specific antibody was used to detect PIF3 in plants. **(C)** Relative PIF3 protein levels in **(B)**. Relative intensities of PIF3 protein bands were estimated from the blots using ImageJ, assuming dark samples as 100%. The intensities of TCTP protein bands were used for normalization. Data represent means ± SD from three independent measurements. **(D)** FR-induced phosphorylation and degradation of PIF3 using transgenic plants co-expressing AsphyA and eGFP-fused PIF3 (PIF3:GFP). Four-day-old dark-grown seedlings were kept in the dark (D) or exposed to pulsed FR light for 1 min (6,000 μmol⋅m^–2^) or 2 min (12,000 μmol⋅m^–2^), and incubated in the dark for 5 min before collecting samples for protein extraction. PIF3:GFP was detected by western blotting using GFP-specific antibody (sc-9996).

Once localized in the nucleus after photoactivation, phytochromes induce rapid phosphorylation of PIF3 preceding the 26S/ubiquitin proteasome-mediated degradation (Al-Sady et al., 2006). In our previous study, we demonstrated that FR light-induced phosphorylation and protein degradation of PIF3 are significantly decreased in transgenic plants of the AsphyA mutants with reduced kinase activity (Shin et al., 2016). As a result, the transgenic plants exhibit hyposensitive responses to FR light. Considering that the K411R and T418V mutants exhibit a higher kinase activity on PIF3 (Figure 1A), we hypothesized that these mutants phosphorylate PIF3 more efficiently, thus inducing a subsequent rapid degradation. To test this hypothesis, we first investigated PIF3 degradation under R and FR light conditions by western blotting with a recently developed PIF3-specific antibody (Choi et al., 2021). As observed in AsA-OX, PIF3 was degraded rapidly in the K411R and T418V plants under R light (Figure 4A). In contrast, under FR light, we detected more PIF3 degradation in the K411R and T418V plants than in AsA-OX. To compare the rates of PIF3 degradation, we further investigated PIF3 protein levels in a time-dependent manner after FR light treatment. As expected, FR induced a mobility shift of PIF3 within 10 min and subsequent degradation progressed rapidly in AsA-OX, whereas no mobility shift and degradation were observed in *phyA-201* plant (Figure 4B). In the K411R and T418V plants, PIF3 phosphorylation and degradation were observed at a much faster pace than those observed in AsA-OX (Figures 4B,C). Within 5 min exposure to FR (5 μmol⋅m^–2^⋅s^–1^), the PIF3 protein band disappeared significantly, and the remaining PIF3 level was comparable to that observed in AsA-OX after 10 min of FR exposure (Figure 4C). These results suggest that the AsphyA mutants with increased kinase activity induce a faster PIF3 degradation than wild-type AsphyA, probably by phosphorylating PIF3 more efficiently.

To verify the relation between PIF3 phosphorylation and degradation more precisely, we used transgenic plants co-expressing AsphyA and eGFP-fused PIF3 (named K411R/PIF3 and T418V/PIF3). The transgenic line co-expressing wild-type AsphyA and PIF3:eGFP (AsA-OX/PIF3), which was used in our previous study (Shin et al., 2016), was included as a control. We selected transgenic lines showing AsphyA and PIF3 protein expression levels comparable to AsA-OX/PIF3 and L*er*/PIF3 (Supplementary Figure 7A). Notably, the hypocotyl lengths of K411R/PIF3 and T418V/PIF3 plants under the cFR light were shorter than those of AsA-OX/PIF3 and L*er*/PIF3, probably indicating the effect of higher kinase activity in the transgenic plants harboring the K411R and T418V mutants (Supplementary Figures 7B,C). Then, we evaluated PIF3 phosphorylation and degradation by applying FRp in the AsphyA and PIF3 co-expression plants. After 1 min FRp treatment (6,000 μmol⋅m^–2^), the mobility shifts of PIF3 were observed clearly in all plants (Figure 4D). With 2 min FRp treatment (12,000 μmol⋅m^–2^), the PIF3 protein bands disappeared in the K411R/PIF3 and T418V/PIF3 plants, whereas the band remained detectable in AsA-OX/PIF3 (Figure 4D). These results showed that the K411R and T418V mutants induced more effective phosphorylation of PIF3, resulting in its faster degradation under FR light.

### HY5 Accumulation in Transgenic Plants

**FIGURE 5:**
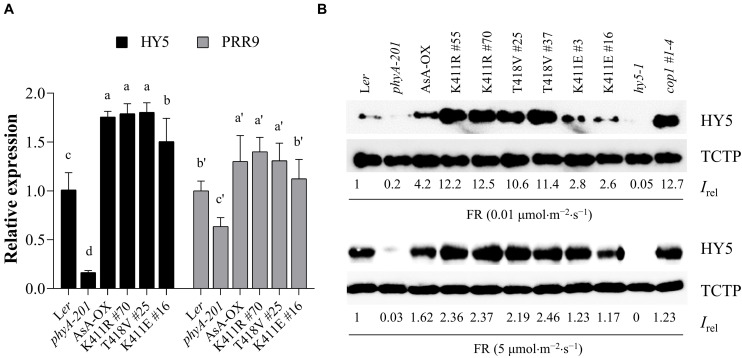
HY5 accumulation in transgenic plants with AsphyA kinase mutants under FR light. **(A)** Quantitative RT-PCR analysis of *HY5* and *PRR9.* Three-day-old dark-grown seedlings were exposed to FR (10 μ mol⋅m^–2^⋅s^–1^) for 1 h, before RNA extraction. The transcript level of *ACT2* was used for normalization, and the relative expression levels were estimated assuming the transcript levels in L*er* as 1. Data represent means ± SD from three independent measurements. Means with different letters are significantly different at *P* < 0.01, using Duncan’s multiple range test. **(B)** Measurements of HY5 accumulation in response to FR light. Seedlings were grown for 4 days under continuous FR (0.01 or 5 μmol.m^–2^.s^–1^) light, and HY5 protein levels were analyzed by western blotting using HY5-specific polyclonal antibody (R1245-1b). The *hy5* and *cop1* mutants were included as negative and positive controls, respectively. Relative intensities (*I*_*rel*_) of HY5 were estimated from the blots using ImageJ, assuming the HY5 band intensity in L*er* as 1. The band intensity of TCTP was used for normalization.

The phytochrome-mediated light signaling in plants is regulated via the transcription of photoresponsive genes (Jing and Lin, 2020). For photomorphogenic development, negative transcriptional factors such as PIFs need to be inactivated, while positive transcriptional factors such as HY5 need to be activated. In particular, HY5 accumulation is important for plant responses to light (Pham et al., 2018b). Thus, we investigated gene expression and protein accumulation of HY5 to examine the relation between the observed FR hypersensitivity and HY5 level in the K411R and T418V plants (Figure 5). Toward this, we first investigated FR-induced expression of *HY5* and *PRR9*, whose expression is induced by phyA (Wolf et al., 2011). Results showed that the transcript levels of both genes were higher in the K411R and T418V plants than in L*er*, but comparable to the level in AsA-OX (Figure 5A). In contrast, the HY5 protein levels were significantly higher (approximately two- to three-fold) in the K411R and T418V plants than in AsA-OX, especially at the FR fluence rate of 0.01 μmol⋅m^–2^⋅s^–1^ (Figure 5B). Notably, the HY5 level in AsA-OX was much higher than in L*er* (4.2 vs. 1), probably owing to the overexpression of monocotyledonous phyA under the 35S promoter in the dicotyledonous plant. In addition, the HY5 level in the K411E plant was notably less than in AsA-OX, but slightly higher than in L*er*, which was consistent with the observed photoresponses (Figures 2, 3). Even at a higher FR fluence rate (5 μmol⋅m^–2^⋅s^–1^), the HY5 levels were maintained at higher level in the K411R and T418V plants than in other control plants (L*er*, AsA-OX, and K411E plants). Taken together, these results suggest that the enhanced FR responses in the transgenic plants are related to the increased HY5 accumulation.

### Regulation of PIF1 by the AsphyA Mutants With Increased Kinase Activity

In our previous study, we used PIF3 to examine the *in vivo* function of phytochrome kinase activity. Thus, we extended our work with PIF1 that negatively regulates chlorophyll biosynthesis and seed germination (Huq et al., 2004; Oh et al., 2004). PIF1 is also regulated by light-mediated phosphorylation and degradation via the ubiquitin/26S proteasome pathway by interacting with photoactivated phytochromes (Shen et al., 2005, 2008). Previously, we showed that AsphyA phosphorylates not only PIF3, but also PIF1 (Shin et al., 2016). Thus, in this study, we initially analyzed PIF1 phosphorylation *in vitro* with the AsphyA mutants and confirmed a higher phosphorylation by the K411R and T418V mutants than by wild-type AsphyA (Supplementary Figure 8). Thus, the present study demonstrated that the mutants show enhanced kinase activities on both PIF3 and PIF1.

**FIGURE 6:**
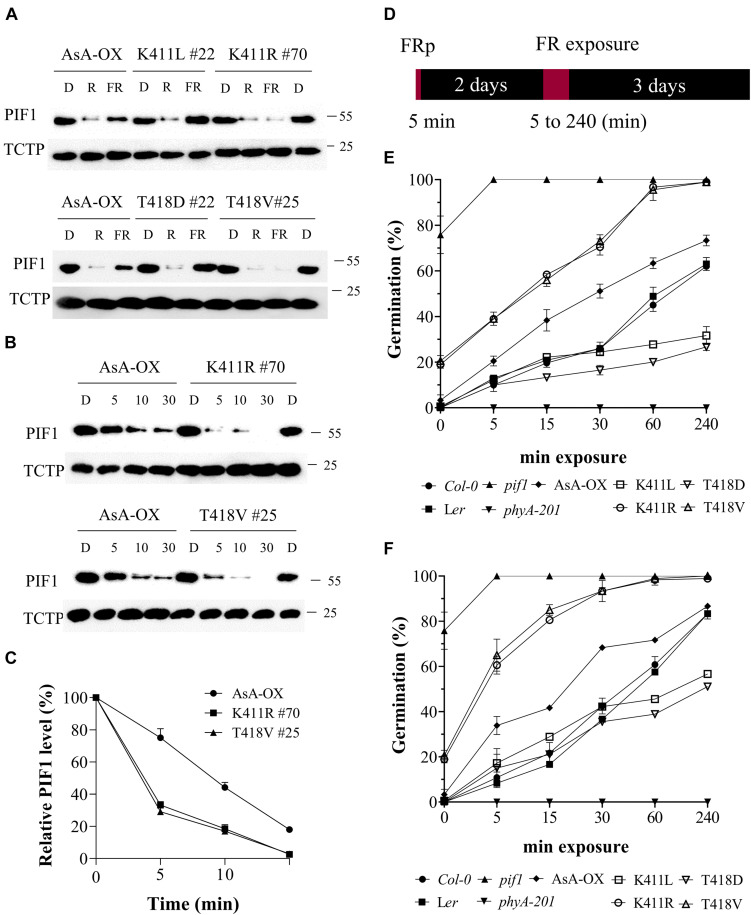
FR-induced degradation of PIF1 and germination analysis of transgenic plants with AsphyA kinase mutants. **(A)** Light-induced degradation of PIF1. Four-day-old dark-grown seedlings were kept in the dark or exposed to either R (20 μmol⋅m^–2^⋅s^–1^) or FR (20 μmol⋅m^–2^⋅s^–1^) light for 5 min, before collecting samples for protein extraction. Loading controls (TCTP) are shown in the lower panels. Transgenic plants of two AsphyA mutants with lower kinase activity (K411L and T418D) were included to compare with those of two mutants with higher kinase activity (K411R and T418V). **(B)** Time-dependent degradation of PIF1 under FR light. Four-day-old dark-grown seedlings were kept in the dark or exposed to FR (20 μmol⋅m^–2^⋅s^–1^) for the time indicated (5, 10, and 30 min), before protein extraction. **(C)** Relative PIF1 protein levels in **(B)**. Relative intensities of PIF1 protein bands were estimated from the blots using ImageJ, assuming dark samples as 100%. The intensities of TCTP protein bands were used for normalization. Data represent means ± SD from three independent measurements. **(D–F)** FR-induced (i.e., phyA-dependent) seed germination. Light scheme for the germination assay is shown in **(D)**. Seeds were irradiated with FR pulse (5 μmol⋅m^–2^⋅s^–1^ for 5 min) and incubated at 21°C for 2 days in the dark. Seeds were then exposed with FR fluence rates of 0.5 μmol⋅m^–2^⋅s^–1^ **(E)** or 5 μmol⋅m^–2^⋅s^–1^ **(F)** for the time indicated (0, 5, 15, 30, 60, and 240 min), and further incubated for 3 days in the dark. Germination percentages were calculated from the number of germinated seeds per total number of plated seeds. In addition to L*er*, *phyA-201* and AsA-OX, Col-0 and *pif1* (Col-0 background) plants were included as controls. Error bar represents SD from three independent measurements.

To analyze phosphorylation and degradation of PIF1 *in vivo*, we produced a PIF1-specific antibody using the method used for the PIF3-specific antibody (Choi et al., 2021). The purified α-PIF1 antibody reacted specifically with only full-length and 71 aa-deleted PIF1 proteins among seven PIFs in Arabidopsis (Supplementary Figure 9A). Moreover, PIF1 was detected in Col-0, but not in *pif1* used as a negative control (Supplementary Figure 9B), and light-induced degradation of PIF1 was also observed in Col-0 (Supplementary Figure 9C). Thus, we used this antibody for studying PIF1 phosphorylation and degradation in the plants. First, we investigated PIF1 degradation under R and FR light using transgenic plants expressing the AsphyA mutants with reduced (K411L and T418D) and increased (K411R and T418V) kinase activities. R light-induced PIF1 degradation, comparable to that in AsA-OX, was observed in all the plants (Figure 6A). However, FR light-induced PIF1 degradation was rather different; almost no FR light-induced PIF1 degradation was observed in the K411L and T418D plants, whereas faint PIF1 bands were detected in the extracts from the K411R and T418V plants. These results further support the positive correlation between the kinase activity of phyA and PIF1 degradation. Moreover, time-dependent FR light-induced PIF1 degradation analysis showed that PIF1 degraded much faster in the K411R and T418V plants than in AsA-OX (Figures 6B,C). Most PIF1 was degraded within 5 min of FR light exposure (20 μmol⋅m^–2^⋅s^–1^) in the K411R and T418V plants, whereas it took more than 10 min in AsA-OX. Thus, these results indicate that the K411R and T418V mutants accelerate PIF1 degradation, probably owing to the increased kinase activity.

PIF1 has been identified as a negative regulator in phytochrome-mediated promotion of seed germination (Oh et al., 2004, 2007; de Wit et al., 2016). Thus, we investigated FR-induced (i.e., phyA-dependent) seed germination using transgenic plants under two FR fluence rates (0.5 and 5 μmol⋅m^–2^⋅s^–1^). In both FR light conditions, the K411R and T418V plants exhibited higher germination frequencies than L*er* and AsA-OX, whereas the K411L and T418D plants showed lower germination percentages (Figures 6D-F and Supplementary Figure 10). As controls, *pif1* seeds germinated regardless of the presence of FR light, whereas *phyA-201* seeds did not germinate under FR light conditions. After 5 min of FRp treatment to turn off the activity of other phytochromes, in addition to *pif1*, some seeds of the K411R and T418V plants germinated (15–20%), whereas the seeds of other plants (L*er*, AsA-OX, and the K411L and T418D plants) showed almost no germination (Figures 6E,F). These germination results may reflect the PIF1 degradation in plants; the faster PIF1 degradation in the K411R and T418V plants increases the germination frequency, whereas the lower PIF1 degradation in the K411L and T418D plants decreases germination. As the degrees of PIF1 degradation are related to the kinase activity of phyA, the present results further support the positive correlation between phytochrome kinase activity and photoresponses in plants.

## Discussion

The molecular mechanism underlying phytochrome-mediated light signaling in plants has long been questioned. In this regard, the present study addresses an important issue, i.e., the protein kinase activity of phytochromes. Phytochromes were initially shown as phosphoproteins and their serine/threonine protein kinase activities were demonstrated *in vitro* (Hunt and Pratt, 1980; Yeh and Lagarias, 1998). Later, the autophosphorylation was suggested to attenuate signals by the accelerated degradation of phyA and the accelerated dark reversion of phyB (Han et al., 2010; Medzihradszky et al., 2013). However, although a few protein phosphatases have been reported (Hoang et al., 2019), protein kinases that phosphorylate phytochromes are not known. To this end, we provided evidence that phytochromes play roles as functional protein kinases in plant light signaling using AsphyA mutants with reduced kinase activity (Shin et al., 2016). In the present study, we provide further evidence using AsphyA mutants with increased kinase activity.

In our previous study, three AsphyA mutants with reduced kinase activity were isolated based on the analysis of site-mutants within a putative ATP binding region (403–426 aa), especially on K411, T418, and D422 (Shin et al., 2016). As K411L, T418D, and D422R mutants showed reduced kinase activities, we mutagenized these sites further. Although recombinant proteins of some mutants (especially D422 site-mutants) could not be expressed sufficiently to analyze their photochemical and biochemical properties, we isolated two AsphyA mutants, K411R and T418V, with increased kinase activity (Figure 1A and Supplementary Figure 2A). Using purified recombinant proteins of these mutants, we confirmed that their photochemical and biochemical properties were similar to those of wild-type AsphyA (Figures 1C,D and Supplementary Figure 1). Subsequently, as shown in our previous study (Shin et al., 2016), the photoaffinity labeling assays confirmed a higher ATP-binding affinity in the K411R and T418V mutants than in wild-type AsphyA (Figure 1B and Supplementary Figure 3). Thus, the mutants phosphorylated PIF3 and PIF1 more efficiently than wild-type AsphyA (Figure 1A and Supplementary Figure 8B). To the best of our knowledge, this is the first report on phytochrome mutants with increased kinase activity.

To extend our study on the functional roles of phytochrome kinase activity in plant light signaling, we generated and analyzed transgenic *phyA-201* plants expressing the K411R and T418V mutants and compared them to control plants, such as L*er*, AsA-OX, and the transgenic plant with the K411E mutant that showed kinase activity similar to wild-type AsphyA. Overall, the K411R and T418V plants were hypersensitive to FR light, as demonstrated by shorter hypocotyls, more opened and expanded cotyledons, increased anthocyanin accumulation, and enhanced blocking of greening (Figures 2, 3A,B, and Supplementary Figure 4). In the K411E plant, the FR responses were less than those of AsA-OX, which were more similar to L*er*. This reduced FR response may be due to the changes in absorption spectra to a shorter wavelength (i.e., 3–4 nm blue-shifted). Previously, transgenic plants with a 6 nm blue-shifted mutant (M549T) were approximately 100-fold less sensitive to FR light than the control plants (Maloof et al., 2001). Thus, the blue-shifted K411E mutant would be less effective in phyA function than wild-type AsphyA, resulting in reduced FR responses of the transgenic plants. In the case of the phenotypes of adult plants, the K411R and T418V mutants were as active as wild-type AsphyA, but not hyperactive (Figures 3C,D). This may reflect the light conditions used in the analysis; FR light was used for the analysis of seedlings, whereas fluorescent WL was used for the analysis of adult plants. In addition, the K411R and T418V mutants exhibited faster light-dependent degradation than wild-type AsphyA (Supplementary Figure 6), which may contribute a reduction of phyA function in plants under WL. Collectively, the present study suggests that the K411R and T418V mutants are hyperactive under FR light conditions, although the hyperactivity is not apparent under WL conditions probably due to their rapid protein degradation.

To elucidate why the K411R and T418V mutants are hyperactive in plants, we performed a series of experiments to analyze the phyA function in plants and made certain observations. First, the light-dependent nuclear localization of K411R and T418V was comparable to that of wild-type AsphyA (Supplementary Figure 5). Second, the light-dependent protein degradation of K411R and T418V was faster than that of wild-type AsphyA (Supplementary Figure 6). This faster protein degradation of the mutants might be caused by the increases in autophosphorylation due to the increased kinase activity (Supplementary Figure 8B), which is consistent with the previous result that the autophosphorylation site-mutants of AsphyA were more stable, with a slow protein degradation, than wild-type AsphyA (Han et al., 2010). Third, *in vivo* PIF3 phosphorylation and the subsequent degradation in response to FR light were significantly faster in the K411R and T418V plants than in AsA-OX (Figure 4). PIF3 is a well-known negative regulator of phytochrome signaling, and plays key roles in preventing photomorphogenesis by promoting skotomorphogenesis (Pham et al., 2018a). Thus, the faster PIF3 degradation by the K411R and T418V mutants corelates well with the hypersensitive responses to FR light. In this study, we detected both endogenous PIF3 and exogenously expressed eGFP-fused PIF3 proteins by western blotting with PIF3-specific and GFP-specific antibodies, respectively. Under our experimental conditions, endogenous PIF3 proteins exhibited multiple slow-migrating PIF3 proteins probably due to hyperphosphorylation before subsequent degradation (Figures 4A,B), while eGFP-fused PIF3 proteins showed more clear band shifts, but not multiple slow-migrating proteins (Figure 4D). This difference may be caused by the eGFP fusion to the C-terminus of PIF3, indicating that the eGFP-fusion construct is useful to investigate PIF3 phosphorylation. Fourth, we extended the *in vivo* phosphorylation and degradation assays to PIF1. Similar to the results of PIF3, the FR-induced phosphorylation and degradation of PIF1 were faster in the K411R and T418V plants than in AsA-OX (Figures 6A–C). In contrast, the FR-induced phosphorylation and degradation of PIF1 significantly decreased in the transgenic plants expressing AsphyA mutants with reduced kinase activity (K411L and T418D). Therefore, these results demonstrate that not only PIF3, but also PIF1 is regulated in plants by the kinase activity of phyA.

PIF1 is a well-known major negative regulator of light-induced seed germination (Oh et al., 2004; Yang et al., 2020); *pif1* plants show light-independent seed germination (Supplementary Figure 10). During the phyA-dependent seed germination analysis using FR light treatment, we also found light-independent seed germination in the K411R and T418V plants, although the germination percentage (∼20%) was lower than that in *pif1* (∼80%) (Figures 6E,F). Moreover, the K411R and T418V plants showed higher germination frequencies under FR light conditions than control plants (L*er*, AsA-OX, and K411E plant), whereas the germination percentages of the K411L and T418D plants were lower. Therefore, these results suggest that light-dependent seed germination is also regulated by the kinase activity of phytochromes.

In general, plant light signaling can be modulated via transcriptional regulation with negative and positive factors. Among the positive regulators, HY5 expression and accumulation directly correlates with the extent of photomorphogenic development (Gangappa and Botto, 2016; Pham et al., 2018b). Thus, we analyzed the expression and protein accumulation of HY5, and found a higher HY5 accumulation in the K411R and T418V plants (Figure 5). In conjunction with the results of PIF3 and PIF1, these data suggest that the K411R and T418V mutants are more active than wild-type AsphyA not only by accelerating the degradation of PIFs but also by inducing the accumulation of HY5. However, at this point, the molecular mechanisms responsible for the increased accumulation of HY5 by these mutants could not be explained fully. However, a couple of hypotheses can be speculated. For example, increased HY5 accumulation in *pifq* plant (Xu et al., 2014) suggests that the degradation of PIFs may lead to a higher HY5 accumulation. In addition, the abundance of HY5 is shown to be regulated by COP1-SPA complexes (Osterlund et al., 2000; Podolec and Ulm, 2018) and photoactivated phytochromes induce the dissociation of these complexes (Lu et al., 2015; Sheerin et al., 2015). Thus, phytochromes negatively regulate the COP1-SPA complexes, resulting in HY5 accumulation to promote photomorphogenic development in plants. Although our results showed that the K411R and T418V mutants degraded PIF3 and PIF1 rapidly, which may have contributed to the high HY5 accumulation in the transgenic plants, we could not rule out the possibility that the mutants also regulate the COP1-SPA complexes differently from wild-type AsphyA. Thus, further studies are necessary to elucidate the molecular mechanisms underlying the high accumulation of HY5 in the K411R and T418V plants.

Collectively, we obtained AsphyA mutants with increased kinase activity and demonstrated the increased phosphorylation and degradation of PIF3 and PIF1 by these mutants *in vitro* and *in vivo*, resulting in hypersensitive responses of their transgenic plants to FR light, such as shorter hypocotyls and higher germination frequencies than in control plants. In addition, we also found an increased accumulation of HY5 in the transgenic plants. Therefore, the present study suggests a positive relationship between the kinase activity of phytochromes and photomorphogenic responses in plants. However, we could not rule out the importance of other kinases that have been reported to phosphorylate PIFs, such as CK2, BIN2, PPKs, and SPA1 (Bu et al., 2011; Ling et al., 2017; Ni et al., 2017; Paik et al., 2019). This is because hyperphosphorylation is necessary for the efficient degradation of PIFs in plants, but phytochromes alone are not enough to mediate the multiple phosphorylation of PIFs (Shin et al., 2016; Hoang et al., 2019). Thus, other kinases might be necessary to act together with phytochromes for the efficient regulation of PIFs. In this regard, it is notable that both PPKs and SPA1 are shown to interact with not only PIF3 and PIF1, respectively, but also with phytochromes (Ni et al., 2017; Paik et al., 2019). These results suggest a possible co-action of phytochromes and other kinases (PPKs and SPA1) for the hyperphosphorylation of PIFs in plants. It is also noteworthy that PPKs may not function as kinases for PIF3 under FR light because *ppk123* mutant did not show any phenotypes under cFR condition (Ni et al., 2017). Therefore, further studies await for answering how kinase activities of phytochromes and other kinases regulate the phosphorylation of PIFs under different light conditions.

## Data Availability Statement

The original contributions presented in the study are included in the article/Supplementary Material, further inquiries can be directed to the corresponding authors.

## Author Contributions

Y-JH and J-IK designed the project. QH, J-YC, D-MC, and A-YS performed the experiments. QH, JK, Y-JH, and J-IK analyzed the data and discussed about the results. QH, Y-JH, and J-IK wrote the manuscript. All authors approved the manuscript.

## Conflict of Interest

The authors declare that the research was conducted in the absence of any commercial or financial relationships that could be construed as a potential conflict of interest.

## Publisher’s Note

All claims expressed in this article are solely those of the authors and do not necessarily represent those of their affiliated organizations, or those of the publisher, the editors and the reviewers. Any product that may be evaluated in this article, or claim that may be made by its manufacturer, is not guaranteed or endorsed by the publisher.

## Supplementary Material

Supplementary Figure 1 Purified AsphyA mutants used in this study.

Supplementary Figure 2 Kinase activity assay of AsphyA site-mutants with PIF3 as a substrate.

Supplementary Figure 3 Photoaffinity labeling of AsphyA with 2-N3-ATP-biotin-long chain-hydrazone (2-azido-ATP) to represent ATP-binding affinity.

Supplementary Figure 4 Transgenic *phyA-201* plants expressing AsphyA mutants.

Supplementary Figure 5 Light-induced nuclear localization of AsphyA kinase mutants.

Supplementary Figure 6 Light-induced degradation of AsphyA proteins.

Supplementary Figure 7 Transgenic *phyA-201* plants co-expressing AsphyA and eGFP-fused PIF3 (PIF3:eGFP).

Supplementary Figure 8 Phosphorylation of PIF1 by AsphyA kinase mutants.

Supplementary Figure 9 Production of PIF1-specific polyclonal antibody.

Supplementary Figure 10 FR-induced (i.e., phyA-dependent) seed germination assays.

Supplementary Table 1 Sequences of the primers used in the present study.

Supplementary Table 2 Calculation of the nuclear/cytoplasmic ratios of eGFP-fused AsphyA signals.
