# Supplementary material for: Protein Kinase Activity of Phytochrome A Positively Correlates With Photoresponses in Arabidopsis
Source: Front Plant Sci. 2021 Jul 30;12:706316. doi: 10.3389/fpls.2021.706316 (PMC8362889; doi:10.3389/fpls.2021.706316)
Supplement: Supplementary Figure 1 — Purified AsphyA mutants used in this study. [file Data_Sheet_1.PDF]

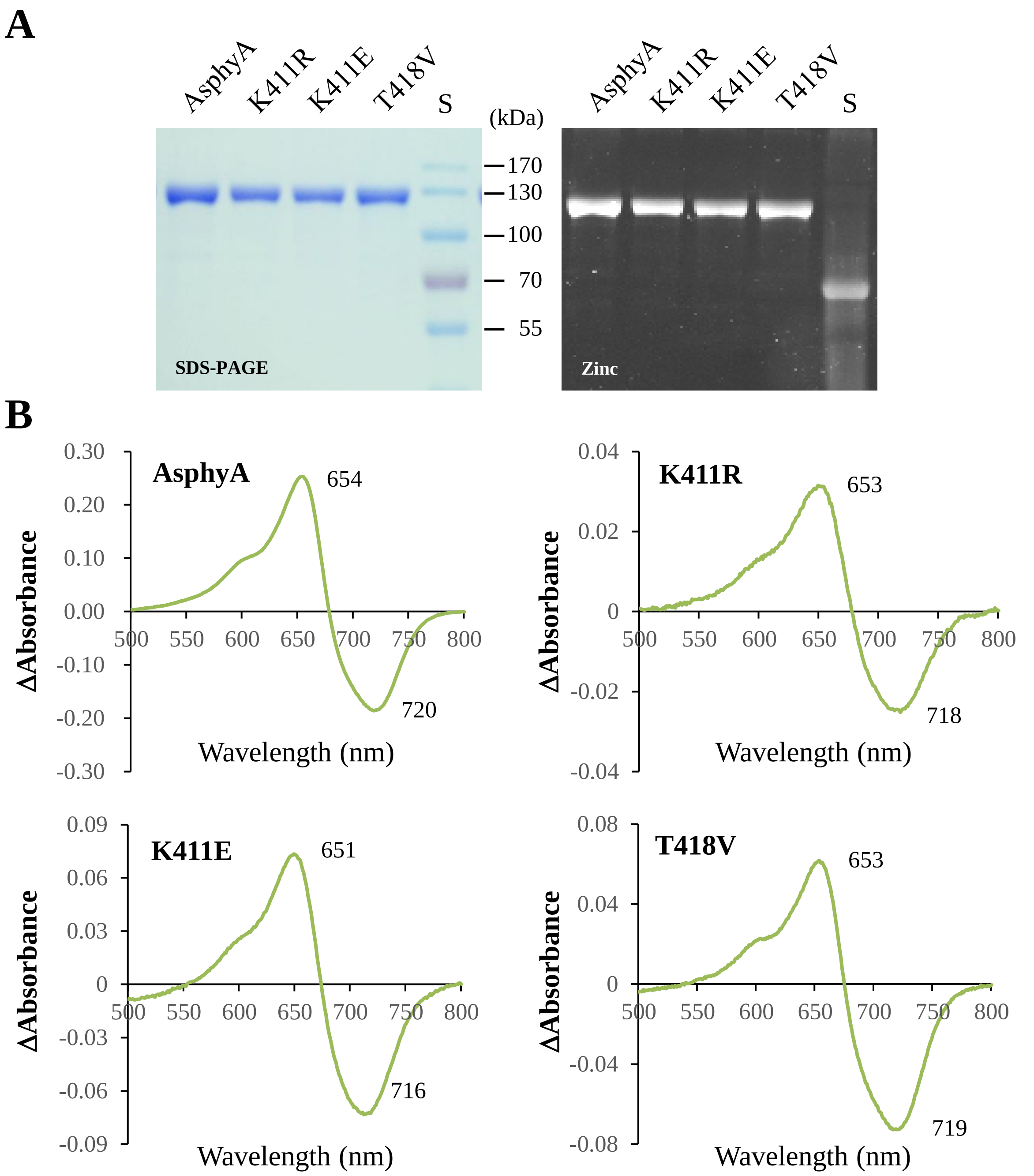

Supplementary Figure S1. Purified AsphyA mutants used in this study. (A) SDS-polyacrylamide gel (SDS-PAGE) and zinc fluorescence (zinc) images of purified recombinant proteins. AsphyA proteins were expressed and purified using the *Pichia pastoris* expression system and streptavidin affinity chromatography, respectively. PageRuler prestained protein ladder (Fermentas) was included in the last lane of the gel (i.e., lane S). **(B)** Difference spectra of the purified AsphyA proteins. The difference spectra (ΔAbsorbance) were obtained by subtracting the Pfr absorption spectra from the Pr absorption spectra. Maximum absorbance peaks (λ_{max}) are labeled as numbers (nm) in the graphs.

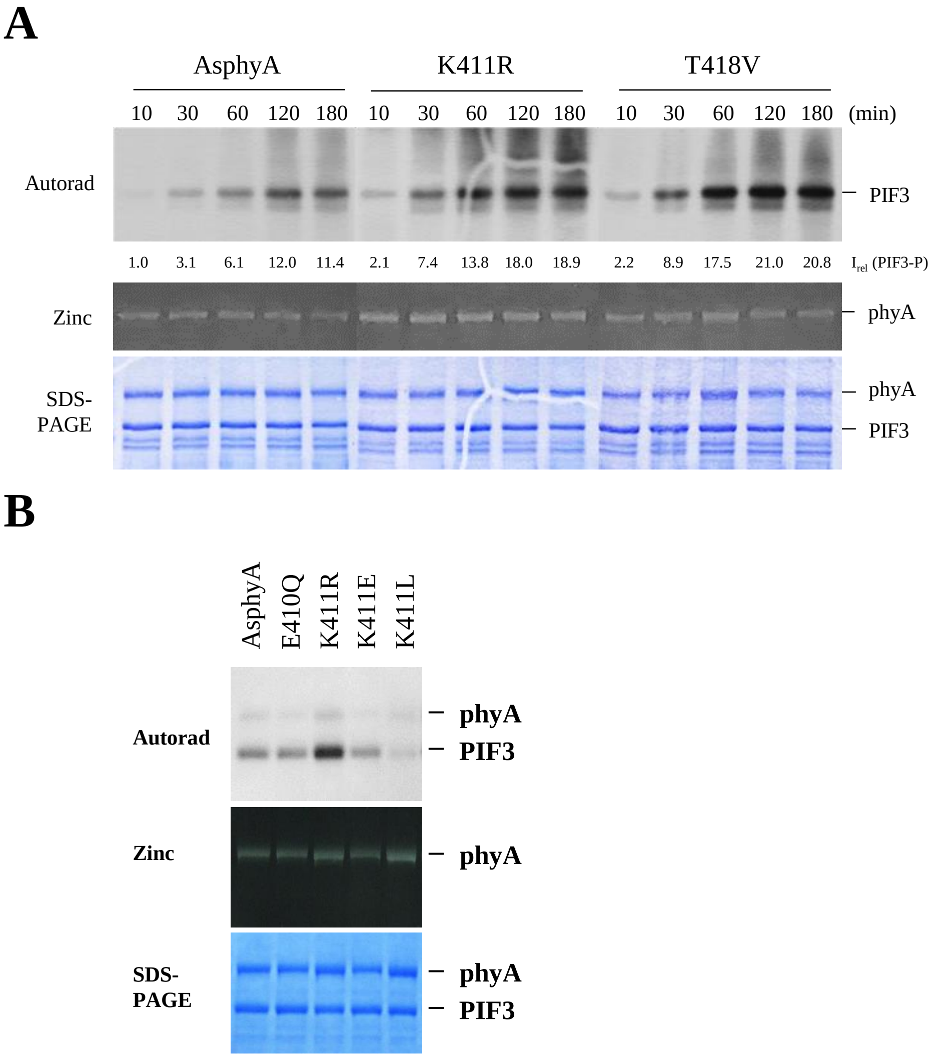

Supplementary Figure S2. Kinase activity assay of AsphyA site-mutants with PIF3 as a substrate. (A) Time-dependent phosphorylation analysis. 1.0 μg of each AsphyA (the Pfr form) and GST/strep-fused PIF3 was used for the assays. Autoradiogram (Autorad), zinc fluorescence (Zinc) and Coomassie-stained gel (SDS-PAGE) are shown. In addition, relative intensities of phosphorylated PIF3 bands [I_{red} (PIF3-P)] were estimated from the Autorad films using ImageJ, assuming the intensity of PIF3-P by AsphyA at 10 min as 1. **(B)** Kinase assays of different AsphyA mutants. E410Q and K411L mutants were reported previously (Shin et al., 2016), while K411E and K411R mutants were newly generated in this study.

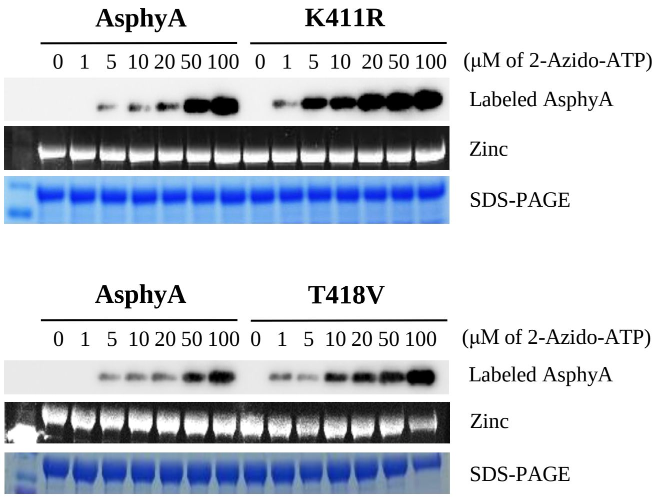

Supplementary Figure S3. Photoaffinity labeling of AsphyA with 2-N_3-ATP-biotin-long chain-hydrazone (2-azido-ATP) to represent ATP-binding affinity. 2.0 µg of full-length AsphyA protein (the Pfr form) with a ten-amino acid streptavidin affinity-tag at the C-terminus was preincubated in a photoaffinity labeling buffer containing unlabeled-ATP for 30 min on ice, and 2-azido-ATP was added at the indicated concentrations. After incubation for an additional 5 min on ice, samples were irradiated with UV light at 254 nm for 90 s and the 2-azido-ATP labeled proteins were detected using avidin-HRP. The zinc fluorescence (Zinc) and Coomassie-stained gels (SDS-PAGE) show the chromophore ligation and the loading controls, respectively.

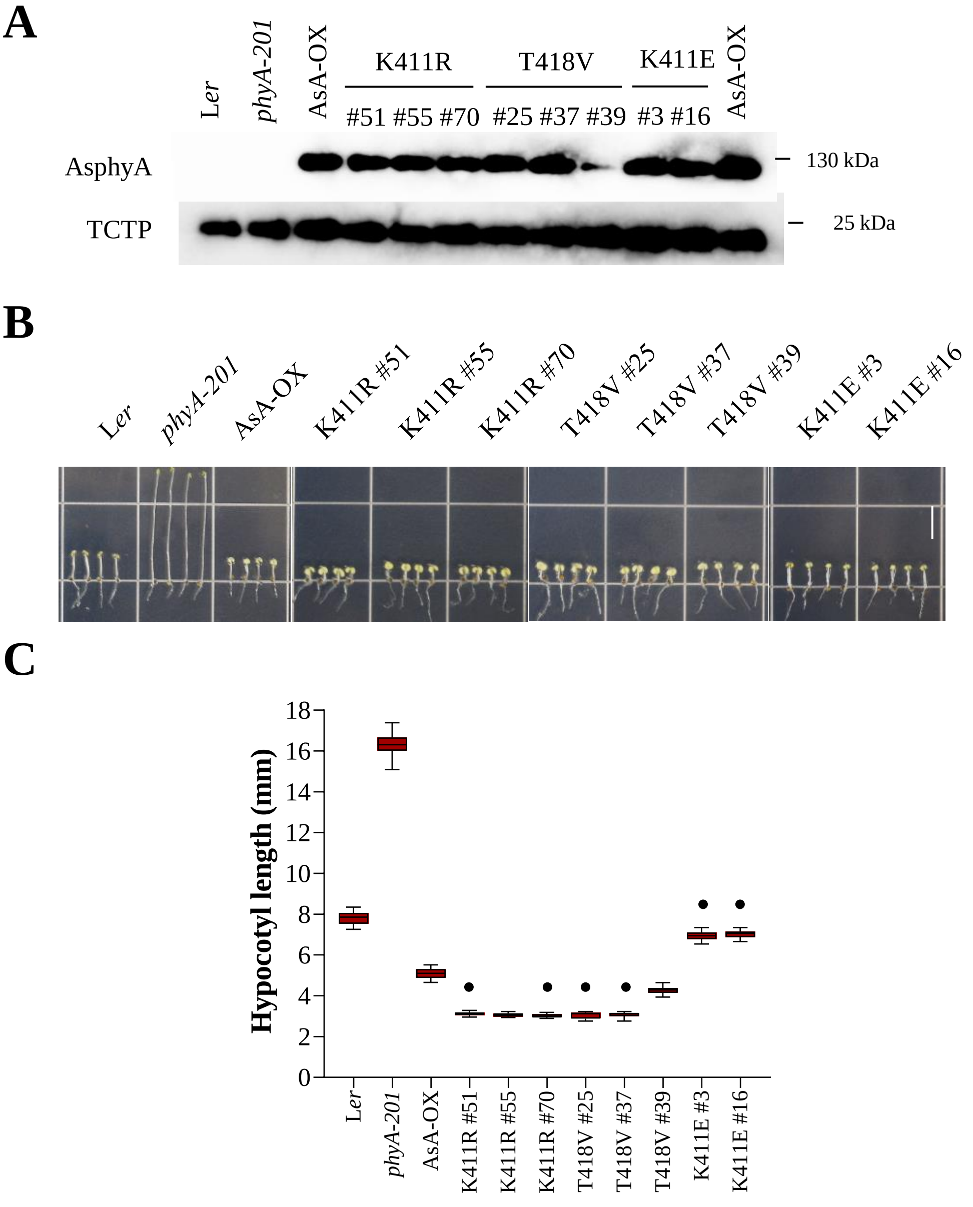

Supplementary Figure S4. Transgenic *phyA-201* plants expressing AsphyA mutants. (A) Immunoblot analysis showing AsphyA protein levels in transgenic plants. L*er*, wild-type Arabidopsis; *phyA-201*, phyA-deficient Arabidopsis (L*er* ecotype); AsA-OX, transgenic *phyA-201* overexpressing wild-type AsphyA; K411R, T418V, and K411E, transgenic *phyA-201* lines with the corresponding AsphyA mutants. Numbers represent independent homozygous transgenic lines. AsphyA-specific (oat25) antibody was used to detect AsphyA, and TCTP (Arabidopsis translationally controlled tumor protein; At3g16640) was shown as loading controls. **(B)** Hypocotyl de-etiolation of representative 4.5-d-old seedlings grown under continuous far-red (cFR, 0.5 $\mu mol \cdot m^{-2} \cdot s^{-1}$) light. Scale bar = 5.0 mm. **(C)** Average hypocotyl lengths of seedlings in (B). Data are the means ± SD (*n* ≥ 30). Filled circles indicate the lines selected for further analysis.

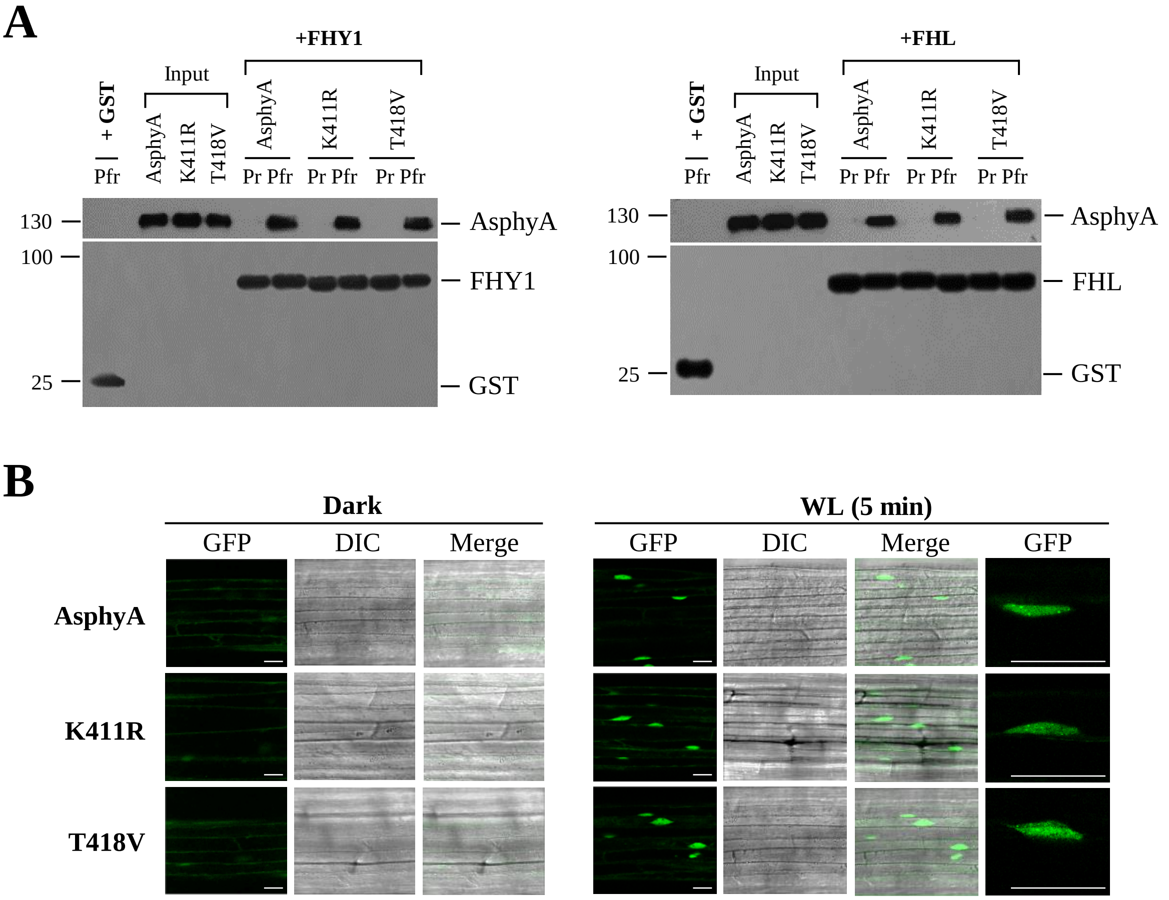

Supplementary Figure S5. Light-induced nuclear localization of AsphyA kinase mutants. (A) In vitro protein–protein interaction analysis of the kinase mutants with FHY1 and FHL. 2 µg of each AsphyA protein (either the Pr or Pfr form) was incubated with 2 µg GST/strep-fused FHY1 (left) or FHL (right) at 4°C, glutathione bead-bound proteins were pelleted, and western blotting was performed using AsphyA-specific (oat25) or GST-specific (sc-138) antibodies. GST was included as a negative control, and ¼ amount of AsphyA protein was loaded to show the input amount. **(B)** Confocal microscope analysis showing the light-induced nuclear localization. Transgenic *phyA-201* plants with eGFP-fused AsphyA constructs were used for these analyses. Hypocotyls of 4-d-old dark-grown seedlings were used directly (Dark) or subjected to white light (WL) for 5 min prior to the GFP fluorescence analysis using a laser scanning confocal microscope. DIC, Different interference contrast. Scale bar = 10 µm.

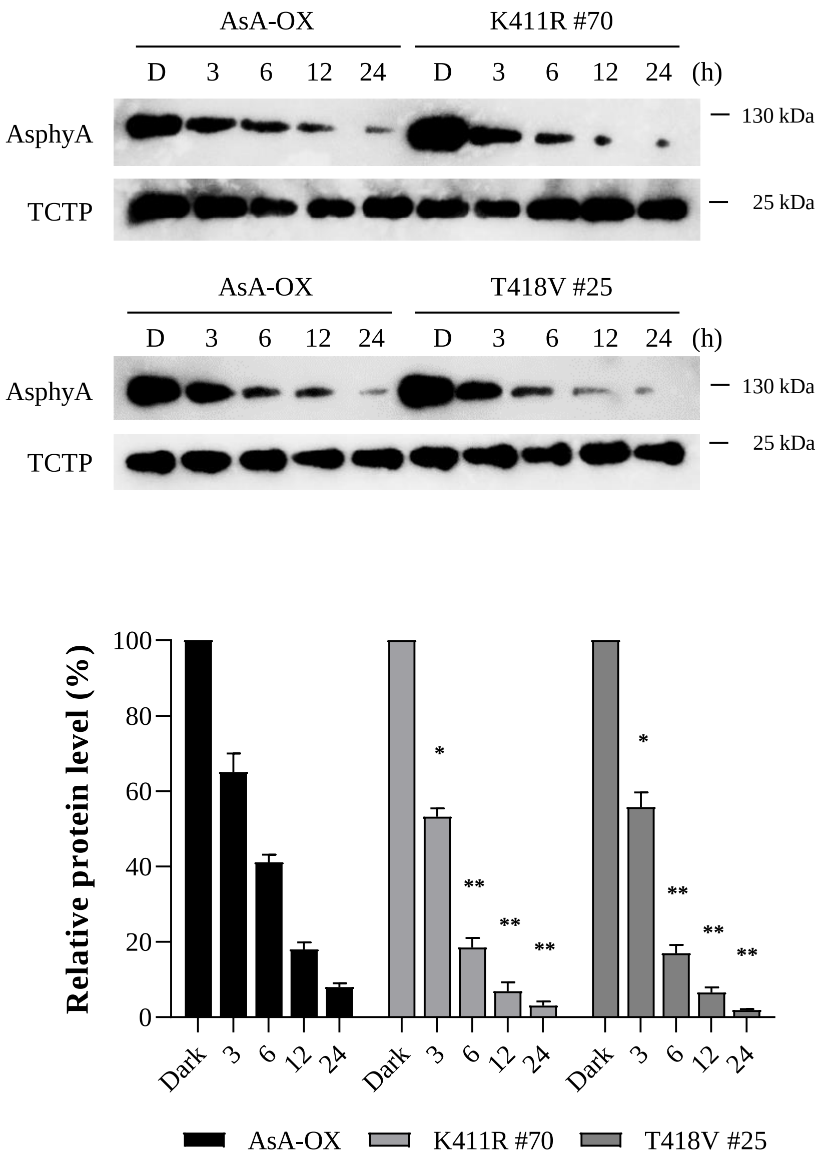

Supplementary Figure S6. Light-induced degradation of AsphyA proteins. (A) Four-day-old dark-grown seedlings of each transgenic plant were exposed to continuous white light (150 μmol·m^{-2}·s^{-1}) for the indicated durations. Total protein extract (60 μg) was used for western blot analysis to detect AsphyA with the oat25 antibody. TCTP shown in the lower panels was used as loading controls.**(B)** Relative protein degradation rates of AsphyA shown in (A). Time-dependent degradation experiments were performed three times and the average percentages compared with the AsphyA protein level in the dark are shown along with SD. Significant changes in comparison to the protein levels of AsA-OX at the same time points are indicated (***P* < 0.01 and **P* < 0.05, Tukey's test).

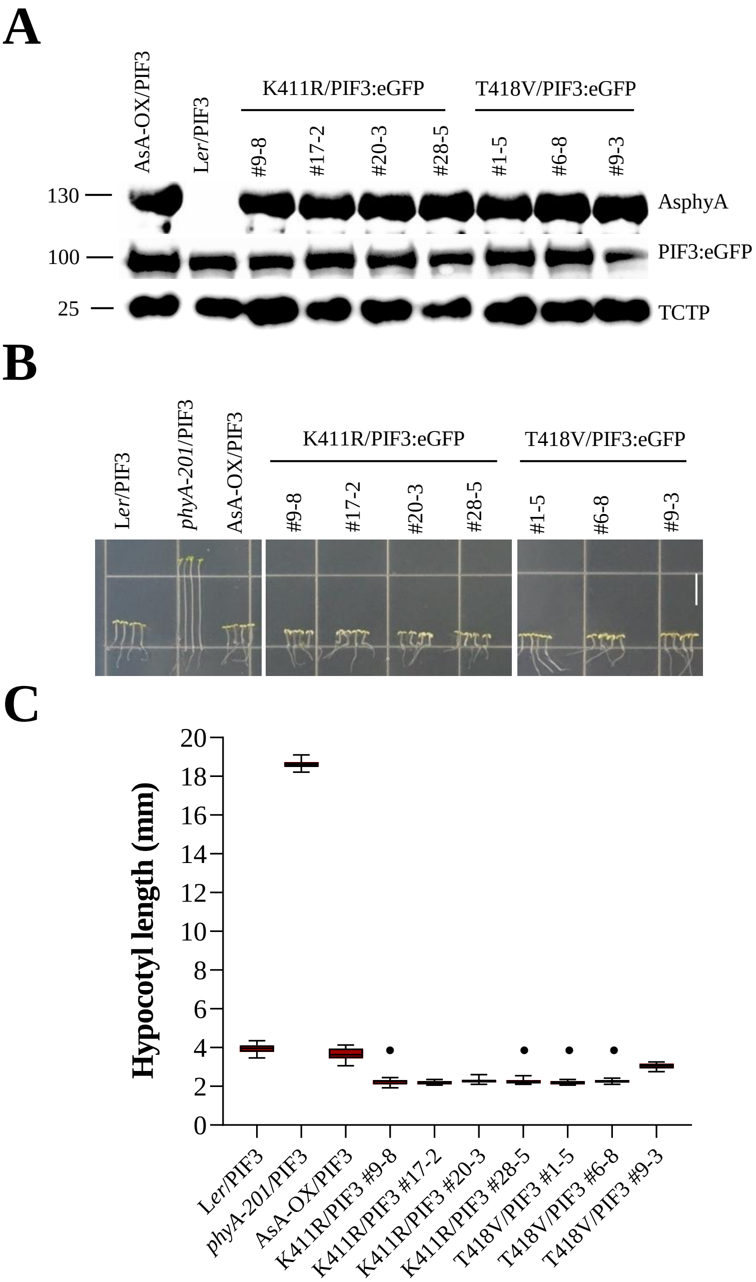

Supplementary Figure S7. Transgenic *phyA-201* plants co-expressing AsphyA and eGFP-fused PIF3 (PIF3:eGFP). (A) Immunoblot analysis showing the protein levels of AsphyA and eGFP-fused PIF3 in dark-grown transgenic seedlings. AsA-OX/PIF3, transgenic *phyA-201* co-expressing wild-type AsphyA and eGFP-fused PIF3 (PIF3:eGFP); L*er*/PIF3, transgenic L*er* expressing PIF3:eGFP; *phyA-201*/PIF3, transgenic *phyA-201* expressing PIF3:eGFP. AsphyA-specific (oat25) and GFP-specific (sc-9996) monoclonal antibodies were used to detect AsphyA and eGFP-fused PIF3, respectively. TCTP was shown for loading controls. **(B)** Hypocotyl de-etiolation of representative seedlings grown for 4.5 d under continuous far-red (cFR, 5 μmol·m^{-2}·s^{-1}) light. Scale bar = 5.0 mm. **(C)** Average hypocotyl lengths of seedlings in (B). Data are the means ± SD ($n \geq 30$). Filled circles indicate the lines selected for further analysis.

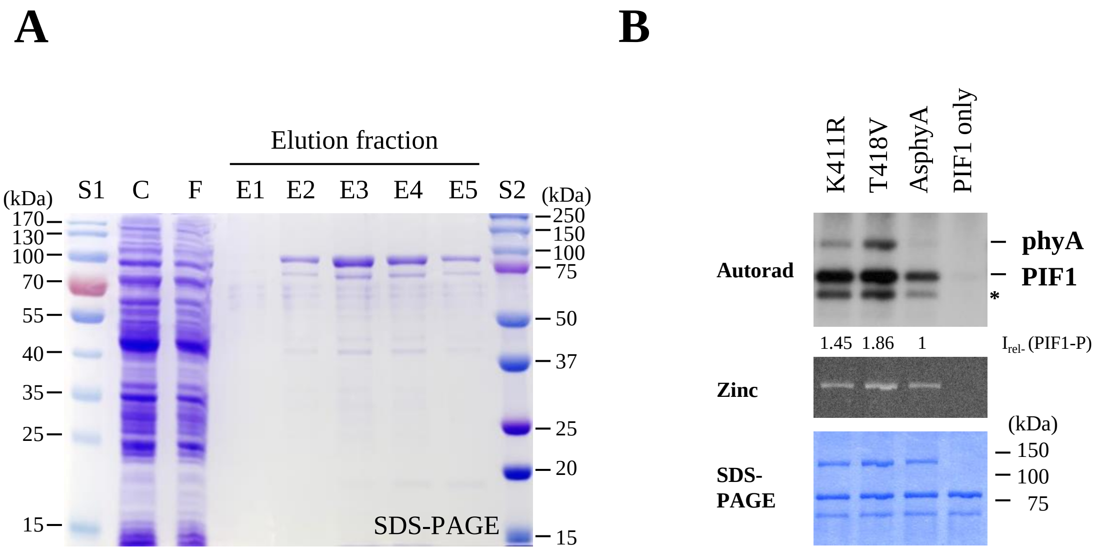

Supplementary Figure S8. Phosphorylation of PIF1 by AsphyA kinase mutants. (A) Recombinant GST/strep-fused PIF1 protein purified from *E. coli*. Glutathione S-transferase (GST) and streptavidin (strep) affinity tags were fused to N- and C- termini of PIF1, respectively. The GST/strep-fused PIF1 protein (79.9 kDa) was purified by the streptavidin affinity chromatography. Lane S1, PageRuler prestained protein ladder (Thermo Fisher Scientific); lane C, crude extract containing GST/strep-fused PIF1 protein; lane F, flow-through fraction from the column; lanes E1-E5, elution fractions of purified GST/strep-fused PIF1; lane S2, Precision Plus Protein™ Dual Color Standards (Bio-Rad). **(B)** Kinase activity assays of AsphyA kinase mutants using PIF1 as a substrate. 1.0 µg of GST/strep-fused PIF1 was added to the reaction with 1.0 µg of full-length AsphyA protein (the Pfr form). Autoradiogram (top), zinc fluorescence (middle), and SDS-PAGE gel (bottom) are shown. Intensities (Irel) of PIF1 phosphorylation are expressed relative to lane 3 (AsphyA). Relative intensities of phosphorylated PIF1 bands [I_{red} (PIF1-P)] were estimated from the Autorad films using ImageJ, assuming the intensity of PIF1-P by AsphyA as 1.

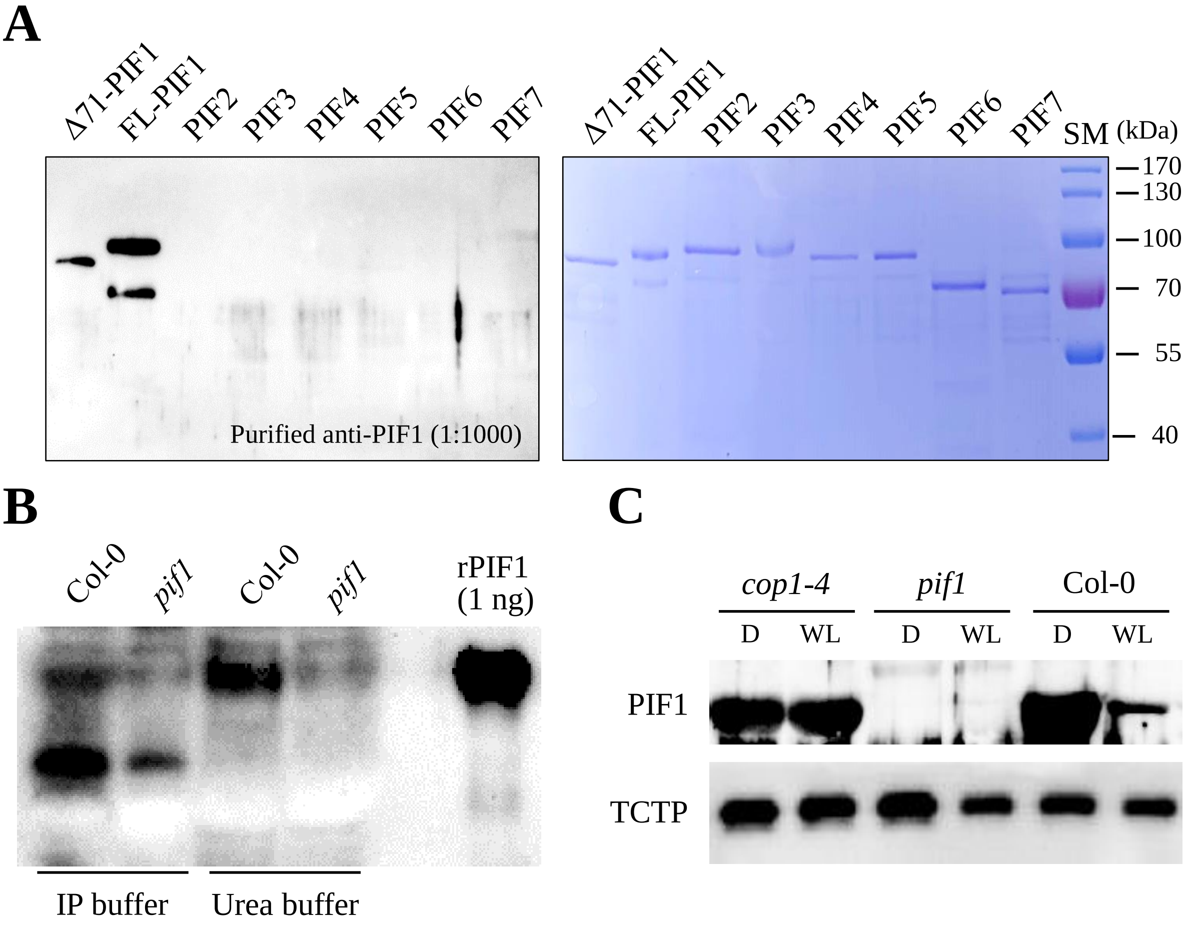

Supplementary Figure S9. Production of PIF1-specific polyclonal antibody. (A) Western blot showing the specificity of anti-PIF1 antibody among Arabidopsis PIFs (PIF1 to PIF7). Seven GST/strep-tagged PIF proteins were expressed in *E. coli* and purified by streptavidin affinity chromatography. In addition, an N-terminal deleted version of PIF1 (Δ71-PIF1; 1-71aa deletion) was included in this western blot performed with the purified anti-PIF1 antibody (1:1,000). **(B)** Western blot with protein extracts of 4-d-old dark-grown Col-0 and *pif1* seedlings. The proteins were extracted with immunoprecipitation (IP) buffer (100 mM Tris-HCl, pH 7.5, 150 mM NaCl, 0.5% NP-40, 1 mM EDTA, 1 mM DTT and protease inhibitors) or urea buffer (100 mM Tris-HCl, pH 7.8, 4 M Urea, and protease inhibitors), and the recombinant PIF1 protein with his affinity-tag (rPIF1) was included as a positive control. **(C)** Western blot showing light-induced degradation of PIF1 in plants. *cop1-4* was included as a negative control to show no light-induced degradation of PIF1. TCTP was used as the loading control.

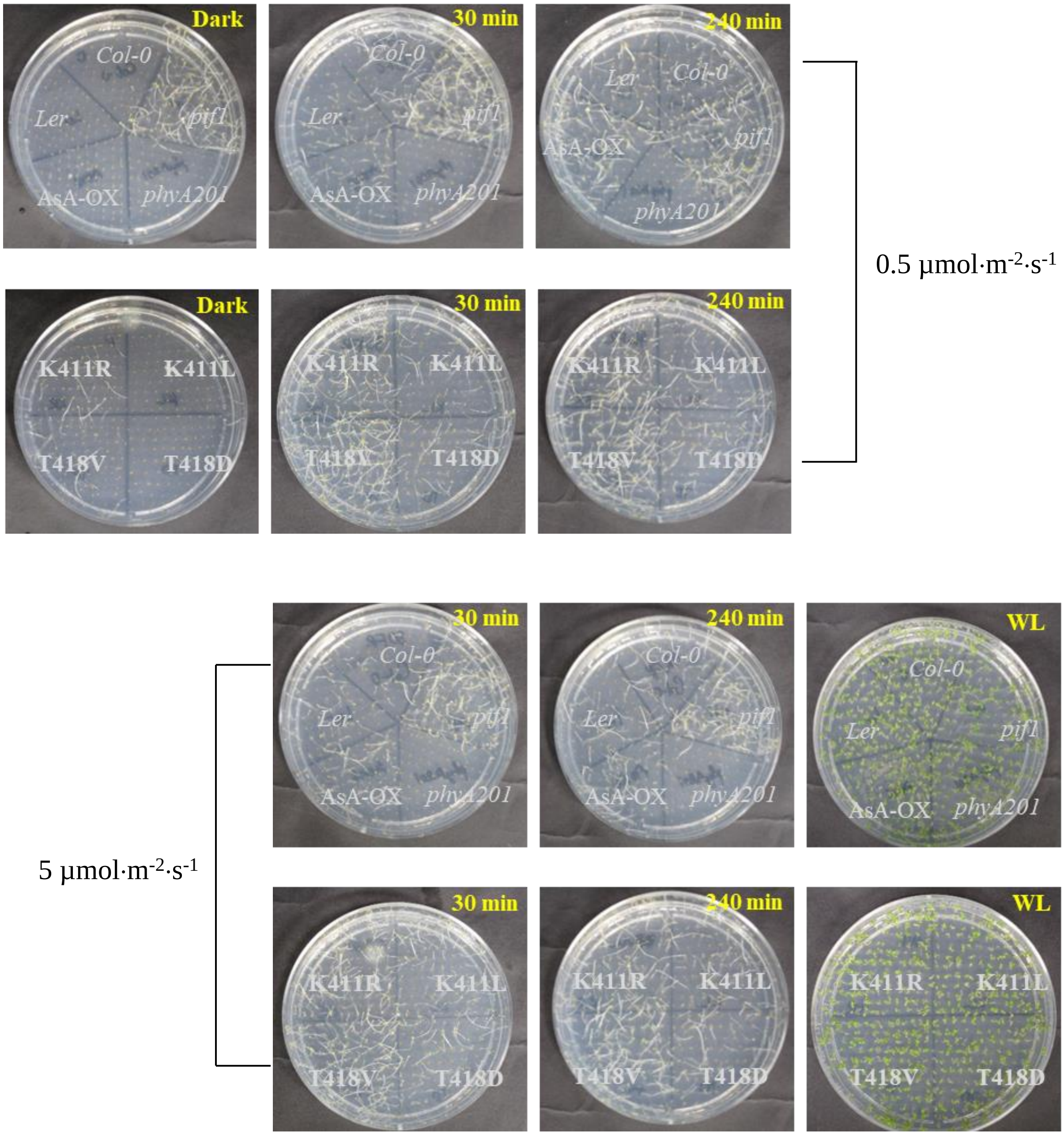

Supplementary Figure S10. FR-induced (i.e., phyA-dependent) seed germination assays. Germination patterns were investigated under 0.5 or 5 µmol·m^{-2}·s^{-1} of FR light, including AsA-OX and transgenic plants with K411R, K411L, T418V, and T418D (L*er* background). In addition, *pif1* (Col-0 background) was included as a control for phyA-independent germination and *phyA-201* was included as a negative control. In addiiton, geminated plates under white light (WL) was shown as controls for germination.

Supplementary Table S1. Sequences of the primers used in the present study.

Oligo name	DNA sequence
For site-directed mutagenesis	
K411E	5'-CAGTTGCGTGAGGAGAACATACTGAAGATG-3'
	5'-CATCTTCAGTATGTTCTCCTCACGCAACTGTTTCTC-3'
K411R	5'-GAAACAGTTGCGTGAGAGGAACATACTGAAGATGC-3'
	5'-TTGCATCTTCAGTATGTTCCTCTCACGCAACTGTTTCTC-3'
T418V	5'-ATACTGAAGATGCAAGTAATGCTCTCTGATATGTTG-3'
	5'-CAACATATCAGAGAGCATTACTTGCATCTTCAGTAT-3'
For real-time PCR analysis	
PRR9	5'-GCCAGAGAGAAGATGCATTGA-3'
	5'-CCTGCTCTGGTACCGAACCTT-3'
HY5	5'-CAAGCAGCGAGAGGTCATCA-3'
	5'-ATCGCTTTCAATTCCTTCTTTGA-3'
ACT2	5'-TCGGTGGTTCCATTCTTGCT-3'
	5'-GCTTTTTAAGCCTTTGATCTTGAGAG-3'

Supplementary Table S2. Calculation of the nuclear/cytoplasmic ratios of eGFP-fused AsphyA signals.[1]

	icn factor[2]	%Nuclei	%Cytoplasm
AsphyA	4.2618	80.9950	19.0050
K411R	4.4192	81.5472	18.4528
T418V	4.1318	80.5136	19.4864

[1]Confocal microscopy images were analyzed with the FIJI Intensity Ratio Nuclei Cytoplasm tool plugin.
[2]icn factor represents the intensities of nuclear/cytoplasmic ratio.
